# Automatic and manual segmentation of the piriform cortex: Method development and validation in patients with temporal lobe epilepsy and Alzheimer's disease

**DOI:** 10.1002/hbm.26274

**Published:** 2023-04-13

**Authors:** David Steinbart, Siti N. Yaakub, Mirja Steinbrenner, Lynn S. Guldin, Martin Holtkamp, Simon S. Keller, Bernd Weber, Theodor Rüber, Rolf A. Heckemann, Maria Ilyas‐Feldmann, Alexander Hammers, for the Alzheimer's Disease Neuroimaging Initiative

**Affiliations:** ^1^ Charité ‐ Universitätsmedizin Berlin Freie Universität and Humboldt‐Universität zu Berlin, Department of Neurology, Epilepsy‐Center Berlin‐Brandenburg Berlin Germany; ^2^ King's College London & Guy's and St Thomas’ PET Centre School of Biomedical Engineering and Imaging Sciences, St Thomas’ Hospital London UK; ^3^ School of Psychology, Faculty of Health University of Plymouth Plymouth UK; ^4^ Department of Pharmacology and Therapeutics, Institute of Systems, Molecular and Integrative Biology University of Liverpool Liverpool UK; ^5^ Department of Neuroradiology The Walton Centre NHS Foundation Trust Liverpool UK; ^6^ Center for Economics and Neuroscience University of Bonn Bonn Germany; ^7^ Institute of Experimental Epileptology and Cognition Research University Hospital Bonn Bonn Germany; ^8^ Department of Medical Radiation Sciences University of Gothenburg Gothenburg Sweden

**Keywords:** Hammers Atlas Database, hippocampal sclerosis, MAPER, mild cognitive impairment, morphometry

## Abstract

The piriform cortex (PC) is located at the junction of the temporal and frontal lobes. It is involved physiologically in olfaction as well as memory and plays an important role in epilepsy. Its study at scale is held back by the absence of automatic segmentation methods on MRI. We devised a manual segmentation protocol for PC volumes, integrated those manually derived images into the Hammers Atlas Database (*n* = 30) and used an extensively validated method (multi‐atlas propagation with enhanced registration, MAPER) for automatic PC segmentation. We applied automated PC volumetry to patients with unilateral temporal lobe epilepsy with hippocampal sclerosis (TLE; *n* = 174 including *n* = 58 controls) and to the Alzheimer's Disease Neuroimaging Initiative cohort (ADNI; *n* = 151, of whom with mild cognitive impairment (MCI), *n* = 71; Alzheimer's disease (AD), *n* = 33; controls, *n* = 47). In controls, mean PC volume was 485 mm^3^ on the right and 461 mm^3^ on the left. Automatic and manual segmentations overlapped with a Jaccard coefficient (intersection/union) of ~0.5 and a mean absolute volume difference of ~22 mm^3^ in healthy controls, ~0.40/ ~28 mm^3^ in patients with TLE, and ~ 0.34/~29 mm^3^ in patients with AD. In patients with TLE, PC atrophy lateralised to the side of hippocampal sclerosis (*p* < .001). In patients with MCI and AD, PC volumes were lower than those of controls bilaterally (*p* < .001). Overall, we have validated automatic PC volumetry in healthy controls and two types of pathology. The novel finding of early atrophy of PC at the stage of MCI possibly adds a novel biomarker. PC volumetry can now be applied at scale.

## Graphical abstract

We describe the first automatic morphometry of the piriform cortex (PC) in healthy controls, patients with temporal lobe epilepsy and Alzheimer's disease. PC atrophy lateralised to the side of hippocampal sclerosis. Early atrophy of PC at the stage of mild cognitive impairment possibly adds a novel biomarker.

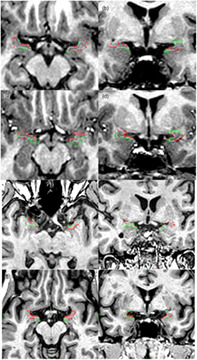

**Abbreviations**
AD: Alzheimer's disease
HS: hippocampal sclerosis
JC: Jaccard coefficient
MAPER: multi‐atlas propagation with enhanced registration
MCI: mild cognitive impairment
PC: piriform cortex
pMCI: progressive mild cognitive impairment
sMCI: stable mild cognitive impairment
TLE: temporal lobe epilepsy

## 1 INTRODUCTION

The piriform cortex (PC) constitutes the largest part of the primary olfactory cortex (Löscher & Ebert, 1996). Situated between the temporal and frontal lobes, it lies rostromedial to the amygdala, covering the fundus of the entorhinal sulcus. It has frontal and temporal lobe parts (Vaughan & Jackson, 2014). Primary functions of PC are olfactory processing and memory coding (Howard et al., 2009; Meissner‐Bernard et al., 2019).

The PC serves as a cortico‐subcortical hub with extensive limbic and cortical connectivity (Pereira et al., 2005; Vaughan & Jackson, 2014). Its projections extend mainly to the periamygdaloid cortex and to the anterior and posterior cortical amygdalar nuclei (Vaughan & Jackson, 2014). For this reason, Gonçalves Pereira et al. (Pereira et al., 2005) subsumed the PC and adjacent regions under the term ‘piriform cortex—cortical amygdala area’. PC forms a network with the perirhinal and entorhinal cortex, connecting it tightly to the limbic system, in particular the hippocampus. Malfunctions in this network develop quickly towards pathological synchronization and seizure spread (Vismer et al., 2015).

Animal studies showed higher susceptibility of the PC to electrical stimulation‐induced epileptic seizures than the hippocampus, amygdala, and entorhinal cortex (McIntyre & Gilby, 2008; Mohapel et al., 2001). Experimentally induced epileptic activity tends to damage PC neurons (Peredery et al., 2000; Roch et al., 2002).

The area tempestas, situated in the anterior (frontal) PC, has been extensively studied in rodents and nonhuman primates. This region is defined based on chemical sensitivity, rather than anatomically: chemoconvulsants induced epileptic seizures at lower concentrations compared with other brain regions (Gale, 1988; Piredda et al., 1985).

Several reports illuminate the role of PC in patients with epilepsy. Using manual segmentations in patients with temporal lobe epilepsy (TLE), Pereira et al. (2005) found PC volumes to be smaller ipsilaterally than contralaterally or than in healthy participants. PC atrophy strongly correlated with hippocampal atrophy/hippocampal sclerosis (HS). A recent study demonstrated progressive atrophy of both ipsilateral and contralateral PC with longer duration of TLE (Iqbal et al., 2022). Resecting a larger portion of PC yielded higher rates of seizure freedom after surgery, suggesting again that the region is involved in seizure generation (Borger et al., 2021; Galovic et al., 2019).

Furthermore, the PC plays crucial roles in other neurological diseases. For example, patients with early‐stage Alzheimer's disease (AD) showed deficits in odour identification, along with impaired response profiles in functional MR of the PC (Li et al., 2010).

Investigators of the cited studies segmented the PC manually, which is time‐consuming. Automatic segmentation would enable studies on larger patient cohorts. Automatic parcellation of the PC based on functional MRI was recently described only in healthy participants (Zhou et al., 2019). Leon‐Rojas et al. (2021) added manual PC segmentation (delineated following the same protocol as in Iqbal et al. (2022)) to the Geodesic Information Flows (GIF) algorithm to create a template for automatic PC segmentation and described the intraoperative use in a single case report.

Here, we developed an automatic segmentation method for the PC on MRI and applied it to patients with atrophy of structures adjacent to the PC. Atrophy was either (1) unilateral (patients with TLE and HS) or (2) bilateral (patients with mild cognitive impairment (MCI) and patients with AD). We added the region to the Hammers Atlas Database (www.brain-development.org) (Faillenot et al., 2017; Gousias et al., 2008; Hammers et al., 2003; Wild et al., 2017) and used the resulting labels to carry out automatic PC segmentations using MAPER (Multi‐atlas propagation with enhanced registration) (Heckemann et al., 2010) in MR images of healthy participants, of patients with TLE and HS, and of patients with MCI or AD. Using quantitative evaluations, we compared and characterized both segmentation methods.

## 2 MATERIALS AND METHODS

### 2.1 Subjects

We segmented the PC manually on the T1‐weighted MR images (voxel sizes ~0.8 mm^3^) of the 30 healthy young adult participants included in the Hammers Atlas Database (www.brain-development.org) (Faillenot et al., 2017; Gousias et al., 2008; Hammers et al., 2003; Wild et al., 2017).

To evaluate our segmentation protocol and the 30 PC segmentations, we used the atlas data set to automatically segment two target data sets obtained in clinical neurological studies.

The first target data set (Keller et al., 2015) consisted of MR images from patients with TLE and unilateral HS who underwent preoperative MR scanning, amygdalohippocampectomy, and postoperative follow‐up at University Hospital Bonn, Germany. An experienced neuroradiologist had identified HS (defined as hippocampal volume loss and internal structure disruption on T1‐weighted scans), and/or hyperintensities on T2‐weighted and FLAIR (fluid attenuated inversion recovery) images. No patient had bilateral HS or extrahippocampal lesions that might have contributed to seizures. HS was confirmed histologically using the International League Against Epilepsy (ILAE) (Scheffer et al., 2017). Scanning took place at Life & Brain Center, Bonn, Germany at 3 Tesla (Magnetom Trio, Siemens, Erlangen, Germany), using an eight‐channel head coil. Morphometric analyses were performed on 3D T1‐weighted MPRAGE (Magnetization Prepared Rapid Gradient Echo) images (160 slices, TR = 1300 ms, TI = 650 ms, TE = 3.97 ms, resolution 1 mm^3^ isotropic, flip angle 10°). Images from 174 participants were available: 39 with right HS (mean age in years 41.6 ± 14.2 (SD) [16–67] (range); 17 female), 77 with left HS (40.7 ± 13.3 [17–70]; 46 female) and 58 healthy participants (40.0 ± 13.9 [18–70]; 34 female). Participants received detailed information about the study and consented in writing. Local ethics guidelines and regulations were followed. The study had been approved by the Ethical Review Board of the Medical Faculty of Bonn.

The second target data set consisted of MR images from the ADNI database (adni.loni.usc.edu; for details of ethical approval, see www.adni-info.org). We downloaded 199 image sets of 151 unique subject IDs of the ‘ADNI cohort 1 Baseline 3 T’. The image sets consist of T1‐weighted baseline MR images acquired at 3 Tesla from patients with AD or MCI, and from healthy controls (voxel sizes 1.73 mm^3^). We used the images labelled as ‘processed’ via each centre's ADNI pipeline, including gradient unwarping (geometric distortion correction) for General Electric (GE) and Siemens scanners and bias correction for GE, Philips, and Siemens scanners (see https://adni.loni.usc.edu/methods/mri-tool/mri-analysis) as recommended by ADNI and to minimise differences between scans. Images and metadata of matching participants (n = 151) were accessed in April 2018. The sample consisted of 33 patients with AD (mean age in years 74.0 ± 8.1 (SD) [57–89] (range); 22 female), 71 with MCI (75.0 ± 8.1 [55–88]; 26 female) and 47 healthy control participants (75.1 ± 3.9 [70–86]; 29 female) (Yaakub et al., 2020). We subdivided the MCI group into progressive (pMCI) and stable MCI (sMCI), based on whether or not their diagnosis record changed to AD at any follow‐up visit up to 36 months. Patients with MCI who converted to AD within 36 months were labelled as progressive MCI (pMCI). Patients whose diagnosis was still recorded as MCI at the last available follow‐up visit were labelled as stable MCI (sMCI).

The work has been carried out in accordance with the code of ethics of the World Medical Association (Declaration of Helsinki).

### 2.2 Segmentation of the PC

#### 2.2.1 Measures of segmentation quality

To measure agreement of tested anatomical labels with reference labels, we relied primarily on the Jaccard overlap coefficient (Jaccard, 1901) (JC). Compared with the Dice coefficient (Dice, 1945), JC numbers are smaller and discriminate better within the range of values encountered in this study (Dice = 2 * JC/[1+ JC]).

To determine volume discrepancy (of which JC is not informative), we used the formula

$$∆V=\frac{{\mathrm{vol}}_{\mathrm{ref}}-{\mathrm{vol}}_{\text{test}}}{{\mathrm{vol}}_{\mathrm{ref}}+{\mathrm{vol}}_{\text{test}}}\cdot 200,$$

where *vol*_*ref*_ is the reference label volume and *vol*_*test*_ is the test label volume. We complemented this directional measure with |∆*V*| (absolute bias), which allows averaging of multiple measurements. Volume discrepancy of left‐sided versus right‐sided labels was used to assess asymmetry.

The manual segmentation protocol minimised the risk of gross shape aberrations or disconnected label components. Automatically generated labels were reviewed visually to ascertain absence of such aberrations. This obviates the need for additional measures of agreement based on surface distances.

#### 2.2.2 Manual segmentations of the PC

Our PC outlining protocol builds on the work of Pereira et al. (2005) and Galovic et al. (2019). According to Pereira et al. (2005), the temporal PC extends caudally from the limen insulae to the opening of the hippocampal fissure, merging medially into perirhinal/entorhinal cortex and laterally into insular cortex. They merged the PC and the cortical amygdaloid nuclei as described above. Their protocol excluded the frontal part of PC because clues to outline its borders on MR images were considered insufficient. In accordance with Vaughan and Jackson (2014), Galovic et al. (2019), and Borger et al. (2021), we included the frontal part of PC. Deviating from Galovic et al. (2019), delineation of PC in the current protocol was terminated in the most rostral slice anterior to the first appearance of hippocampus to avoid any overlap with hippocampal regions. In the outlining protocol, the term ‘piriform cortex (PC)’ refers to this definition. The protocol is provided as a supplement.

The Hammers Atlas Database (www.brain-development.org) has been described previously (Faillenot et al., 2017; Gousias et al., 2008; Hammers et al., 2003; Wild et al., 2017). Briefly, it contains 3D T1‐weighted images of 30 healthy young adults. The images are rotated to position the anterior and the posterior commissure in common axial and sagittal planes (AC‐PC alignment). Separate, spatially corresponding 3D label images contain labels of 95 anatomical regions obtained by manual segmentation. PC segmentation was part of a larger, ongoing initiative to extend the database to 120 regions in total. Using ITK‐SNAP Version 3.8 (Yushkevich et al., 2006), each image was displayed in three orthogonal planes, with the corresponding segmentation added as a colour overlay. After identifying the approximate location of the PC, the most rostral coronal plane intersecting with the PC was selected to draw the first outline. The outlining then proceeded section by section in caudal direction. Existing regions were partially relabelled to create the new PC regions, notably: background (region label 0), amygdala (3, 4), anterior temporal lobe, medial part (5, 6), parahippocampal and ambient gyrus (9, 10), insula anterior pole (92, 93) and posterior long gyrus (20, 21), as well as posterior orbital gyrus (72, 73).

We applied the protocol to patients from the target data sets (20 each, chosen randomly), enabling comparisons of automatically and manually determined labels in pathology.

#### 2.2.3 Intrarater and interrater analysis of manual PC segmentation

We conducted a reliability analysis in test–retest fashion to validate the outlining protocol. Rater 1 (D.S.) segmented the right and left PC twice at an interval of 1 week on the images of five participants from the Hammers Atlas Database.

Additionally, we performed an objectivity analysis with two additional independent raters, M.S. (Rater 2) and L.G. (Rater 3). Both outlined the right and left PC of the same five participants who had been selected for the reliability analysis. Before the first round of segmentations, the second rater and the third rater were only provided with the written outlining protocol (supplement). One week later, the two additional raters independently underwent one practice session, where Rater 2 and Rater 3 practised segmenting the PC under guidance from Rater 1. Images from two participants were used for the practice session; these were not among the five participants used for the previous analysis. Six weeks (Rater 1) or 4 weeks (Rater 2) later, the raters segmented the same five participants a second time.

For both intrarater and interrater analyses, we determined JC, using the first round of segmentations by Rater 1 as the reference.

#### 2.2.4 Automatic segmentation of the PC

Automatic segmentation of the PC was performed using MAPER (Heckemann et al., 2010) (https://github.com/soundray/maper), an application for multi‐atlas‐based brain image segmentation. MAPER implements an ensemble machine learning approach to transfer anatomical knowledge represented in an atlas database to a target image. Each atlas is paired with the target and registered using free‐form deformation (Rueckert et al., 1999), initially considering tissue probability maps and subsequently T1‐weighted images. The resulting geometric transformation is applied to the atlas labels, generating one segmentation label set per atlas in the target space. The individual label sets are then combined into a single output label set using vote‐rule decision fusion (Kittler et al., 1998).

We conducted a leave‐one‐out cross‐comparison analysis to measure agreement (JC, volume error) between MAPER‐generated PC labels and manually generated ones.

Bland–Altman analysis was used to detect or exclude proportional bias on the volume measurements.

We then used MAPER with all 30 atlases to segment the PC in the target data sets. We determined left/right asymmetry for each patient group (TLE with left/right sided HS; AD) and the healthy control groups. For method validation in patients with TLE and those with AD (cf. results Section 3.2), volumes of PC, amygdala, and hippocampus were normalised by intracranial volume (ICV; region volumes divided by ICV, scaled by 10^4^ for ease of reading). ICV was estimated using Pincram (Heckemann et al., 2015) (https://github.com/soundray/pincram).

As a sanity check, volumes of hippocampus and amygdala corrected by ICV were measured in both clinical data sets.

On the random samples from the TLE and AD data sets (20 each; see above) automatic‐to‐manual label agreement was determined. Automatic segmentation was performed in native space. The manual segmentation in 40 patients had been performed on AC‐PC‐aligned images as mandated by the protocol (cf. Section 2.2.2.). Therefore, co‐registration of the automatic to the manual segmentation was performed first, using Statistical Parametric Mapping (SPM12; Wellcome Trust Centre for Neuroimaging; https://www.fil.ion.ucl.ac.uk/spm/software/spm12), employing nearest‐neighbour interpolation for reslicing the atlases.

#### 2.2.5 Statistical analysis

Continuous variables are displayed as mean ± standard deviation and coefficient of variance [CV]. Data were tested for normal distribution using the Kolmogorov–Smirnov test. As the result was nonsignificant, we used parametric tests (two‐sided *t* test and analysis of variance [ANOVA]). Two‐tailed Pearson correlation coefficients were calculated to measure correlation between average PC volume and PC volume difference (Bland–Altman analysis). TOST (two‐one‐sided *t* tests) procedures were carried out to test for equivalence of the volumetry results between scanner types. The significance threshold was set at *p* = .05 before Bonferroni adjustment, which was applied as appropriate. We used IBM SPSS Statistics 24.0 (IBM Corp.) for the analysis.

## 3 RESULTS

### 3.1 Method development

#### 3.1.1 PC volumes and right/left differences

PC was identified and delineated on 7 to 12 slices per image. The mean volume of manual PC labels in the Hammers Atlas Database was 473 mm^3^ ± 94 mm^3^ (CV 20%); for the automatic labels it was 464 mm^3^ ± 66 mm^3^ (CV 14%).

No significant left/right difference in mean PC volumes was evident in the Hammers Atlas Database group. For manual segmentation, mean absolute right/left volume discrepancy was 15 ± 12, CV 81%; *t* = −1.02, *df* = 58, *p* = .31. For automatic segmentation, mean absolute right/left volume discrepancy was 9 ± 7, CV 76%; *t* = −0.74, *df* = 58, *p* = .46.

#### 3.1.2 Reliability and objectivity

The mean intrarater JC (reliability) was 0.70 ± 0.03. Objectivity, as measured by mean interrater JC (Rater 1 vs. Rater 2 or Rater 3) was 0.56 ± 0.04. Mean JC values in the intrarater analysis were similar between the right and left side (0.69 right, 0.71 left; cf. Table 1).

**TABLE 1:** Intrarater and interrater analysis (reliability and objectivity).

		Intrarater	Interrater analysis (post‐training sets)	
	Rater 1 (D.S.)	Rater 1 (D.S.)	Rater 2 (M.S.)	Rater 3 (L.G.)
Right PC				
JC (CV)	‐	0.69 (5%)	0.58 (6%)	0.57 (4%)
Volume in mm^3^ (CV)	497 (11%)	564 (9%)	644 (16%)	485 (7%)
|ΔV| (CV)	‐	13 (44%)	26 (58%)	9 (79%)
Left PC				
JC (CV)	‐	0.71 (3%)	0.53 (12%)	0.57 (7%)
Volume in mm^3^ (CV)	461 (11%)	486 (9%)	586 (11%)	481 (10%)
|ΔV| (CV)	‐	7 (57%)	24 (27%)	7 (64%)

*Note*: Overlap (JC), mean volumes, and mean absolute volume discrepancies (|ΔV|) for PC label pairs in a series of five Hammers Brain Atlas database participants (healthy young adults; same five used for all measurements).

Overlaps in the interrater analysis were lower than in the intrarater analysis for both additional raters, but improved following the practice session. For comparison between Rater 1 and Rater 2, mean JC increased by 0.09 for the right PC (0.58 vs. 0.49) and by 0.12 for the left PC (0.53 vs. 0.41) between the first and second rounds of segmentation. For the comparison between Rater 1 (D.S.) and Rater 3 (L.G.), mean JC improved by 0.25 for the right PC (0.57 vs. 0.32) and by 0.32 for the left PC (0.57 vs. 0.25) after practice.

**FIGURE 1:**
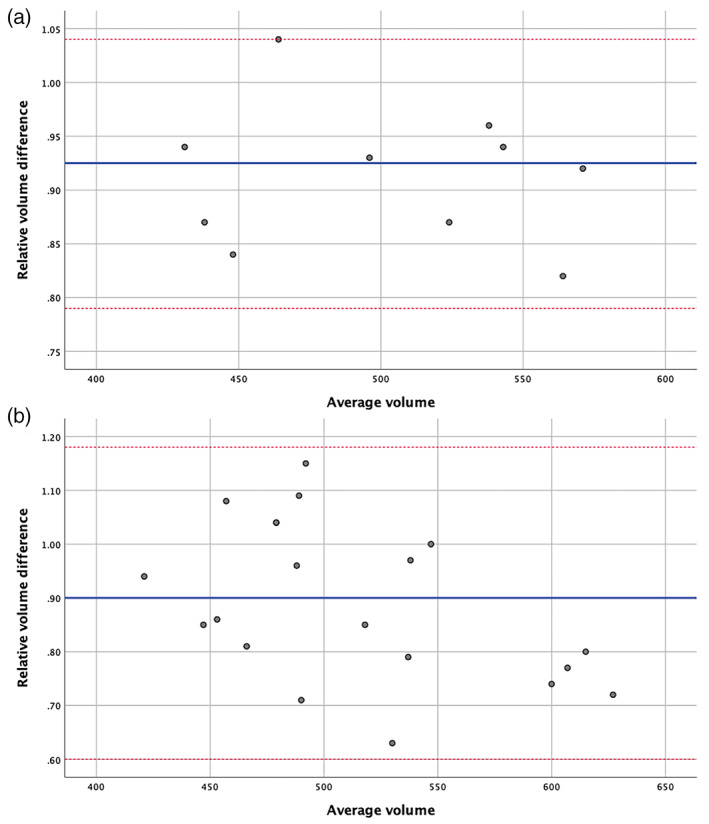
Intrarater reliability analysis and interrater objectivity analysis. Bland–Altman plots illustrating absolute difference in volume of piriform cortex (PC) between (a) first and second segmentation of same rater (intrarater reliability analysis) and (b) between first and second or third rater (interrater objectivity analysis) in five healthy participants (Hammers Atlas Database). Blue lines show the mean difference, red lines show the standard deviation multiplied by 1.96.

Volume discrepancies were similar between the right and left side (Table 1). The relative volume differences in relation to the average of both volumes of a pair of delineations in intrarater and interrater analysis are illustrated in Bland–Altman plots. A degree of inverse‐proportional bias is evident between average PC volume and relative PC volume difference in the interrater objectivity analysis (*r* = −.58, *p* = .008; Figure 1).

#### 3.1.3 Comparison of manual and automatic segmentations

##### Hammers Atlas Database

**FIGURE 2:**
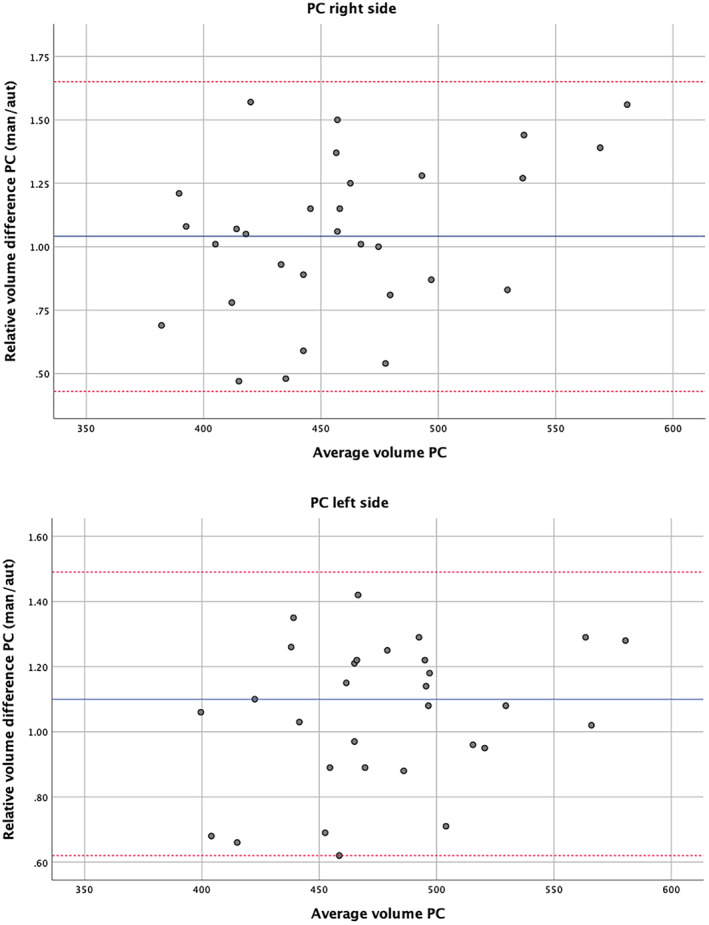
Volume difference between manual and automatic segmentation. Bland–Altman plots illustrating relative difference in volume of piriform cortex (PC) between manual and automatic delineation in 30 healthy participants (Hammers Atlas Database). Blue lines show the mean difference, red lines show the standard deviation multiplied by 1.96.

Manual and automatic segmentations of the PC in the Hammers Atlas Database group overlapped with a mean JC of 0.52 (right) and 0.47 (left) (Table 2). For volume discrepancies, see Table 2. The relative volume differences between automatic and manual segmentations are shown in Bland–Altman plots (Figure 2). They illustrate moderate proportional bias between average volume and volume difference for right PC (*r* = 0.38; *p* = .04). For left PC, no significant correlation was found (*r* = .27; *p* = .15). Likewise, Bland–Altman plots for absolute volume differences (Figure S1) show moderate proportional bias only for the right PC.

**TABLE 2:** Comparison between manual and automatic segmentations.

	Healthy participants (*n* = 30)		Patients with TLE + HS (*n* = 20)		Patients with AD (*n* = 20)	
	Manual	Automatic	Manual	Automatic	Manual	Automatic
Right PC						
JC (CV)	0.52 (13%)		0.41 (20%)		0.35 (27%)	
Volume in mm^3^ (CV)	485 (16%)	470 (12%)	469 (22%)	480 (10%)	345 (32%)	373 (15%)
ICV‐corrected volume (CV)	3.68 (18%)	3.56 (11%)	3.18 (25%)	3.28 (15%)	2.46 (35%)	2.64 (17%)
|ΔV| (CV)	19 (67%)		22 (88%)		27 (83%)	
Left PC						
JC (CV)	0.47 (16%)		0.39 (21%)		0.34 (20%)	
Volume in mm^3^ (CV)	461 (23%)	458 (16%)	373 (30%)	501 (14%)	299 (36%)	332 (23%)
ICV‐corrected volume (CV)	3.48 (22%)	3.45 (14%)	2.52 (27%)	3.45 (18%)	2.13 (39%)	2.35 (25%)
|ΔV| (CV)	25 (79%)		35 (77%)		30 (55%)	

*Note*: Overlap (JC), mean volumes, and mean absolute volume discrepancies (|ΔV|) for PC label pairs in the Hammers Atlas Database, temporal lobe epilepsy with hippocampal sclerosis (TLE + HS), and Alzheimer's disease (AD).

**FIGURE 3:**
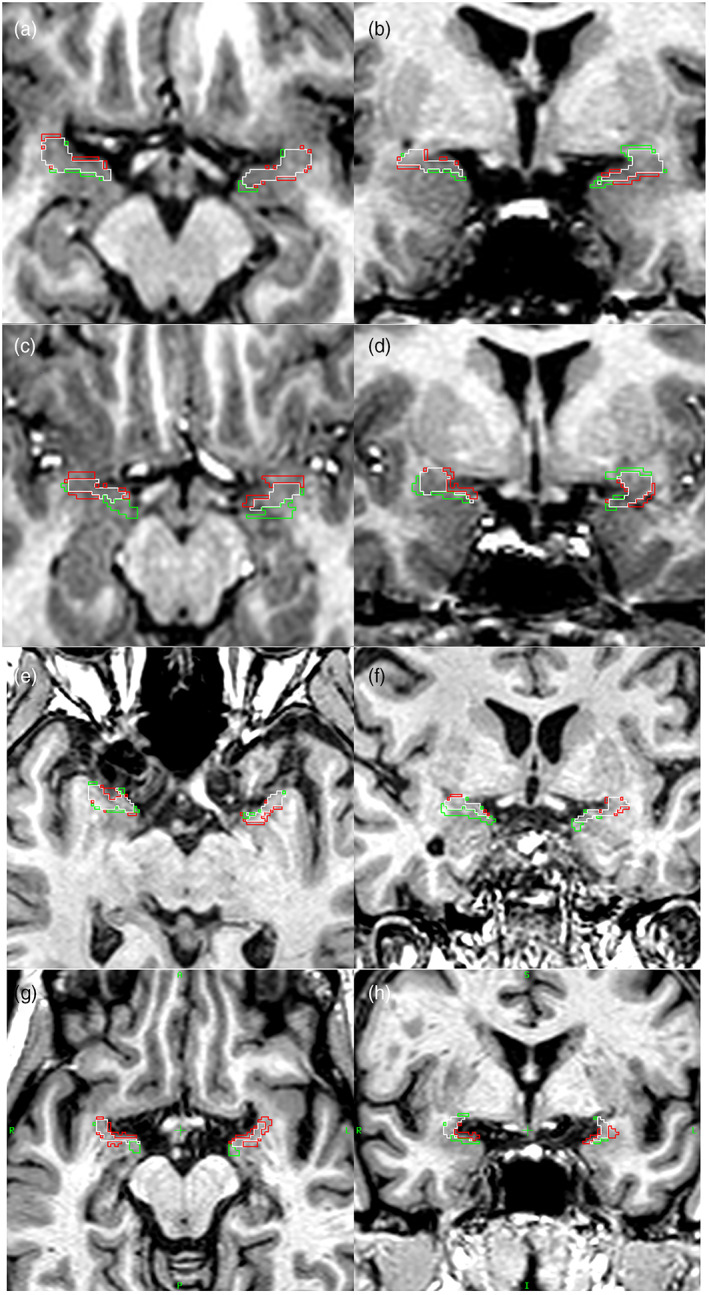
Segmentations of PC. Manual (M) and automatic (a) PC labels, outlined on sections of the corresponding T1‐weighted image in the transverse (Panels a, c, e, g) and coronal (Panels b, d, f, h) planes. Image orientation is identical in all panels (markers in Panels g and h indicate R = right, L = left, A = anterior, P = posterior, S = superior, I = inferior). Outline colours: white indicates label agreement (M ∩ A), red outlines surround regions included by the manual segmentation only (M\A), green outlines surround regions included by the automatic segmentation only (A\M). In Participant 16 of the Hammers Atlas Database (Panels a and b), label agreement is strong (JC right 0.61, JC left 0.56, ∆V right/left = 2/23). In Participant 9 of the Hammers Atlas Database (Panels c and d), label agreement is moderate (JC right 0.36, JC left 0.38, ∆V right/left = 19/1). Panels e and f pertain to a patient with temporal lobe epilepsy with left‐sided hippocampal sclerosis (JC right 0.44, JC left 0.47, ∆V right/left = 36/27), and Panels g and h pertain to a patient with Alzheimer's disease (JC right 0.37, JC left 0.34, ∆V right/left = 27/−20).

Figure 3 shows two examples of a manual/automatic pair of PC labels, taken from the Hammers Atlas Database group, and illustrates the regions of agreement and mismatch within the chosen section.

##### Patients with TLE and patients with AD

In the 20 patients with TLE and HS, on unflipped images (i.e., not considering the lateralization of the epilepsy focus), the agreement between automatic and manual segmentation was slightly lower than in the Hammers Atlas Database group, with a mean JC of 0.41 (right) and 0.39 (left) (Table 2). For the group of 20 patients with AD, a mean JC of 0.35 (right) and 0.34 (left) was found. Volume discrepancy values are shown in Table 2 and volume differences are illustrated in Figure S2. For PC volumes distinguishing between ipsilateral and contralateral sides in a larger patient sample, see the following section. One example of each patient group is illustrated in Figure 3.

#### 3.1.4 Scanner manufacturer

The PC volumes of the ADNI cohort (*n* = 151) differentiated by scanner type are shown in Table 3. The results of the equivalence test (TOST) of the volume measures between scanner types can be found in Table S1. The null hypothesis of nonequivalence can be rejected on the basis of the *p*‐values obtained.

**TABLE 3:** Comparison between scanner types.

	GE (*n* = 23)	Philips (*n* = 50)	Siemens (*n* = 78)
Right PC			
Volume in mm^3^ (CV)	372 (25%)	401 (17%)	385 (20%)
ICV‐corrected volume (CV)	3.00 (23%)	3.20 (16%)	3.11 (20%)
Left PC			
Volume in mm^3^ (CV)	369 (24%)	388 (19%)	350 (19%)
ICV‐corrected volume (CV)	2.98 (21%)	3.10 (19%)	2.83 (20%)

*Note*: Mean volumes and mean volumes normalised by ICV of the ADNI cohort (*N* = 151), differentiated by scanner manufacturer.

### 3.2 Biological method validation and findings in patient groups

There was no significant association between age and ICV‐corrected PC volume in healthy controls (NC) in the TLE NC group (*r* = −.062, *p* = .64, *n* = 58) or in the ADNI NC group (*r* = −.134, *p* = .37, *n* = 47).

#### 3.2.1 Patients with TLE

**FIGURE 4:**
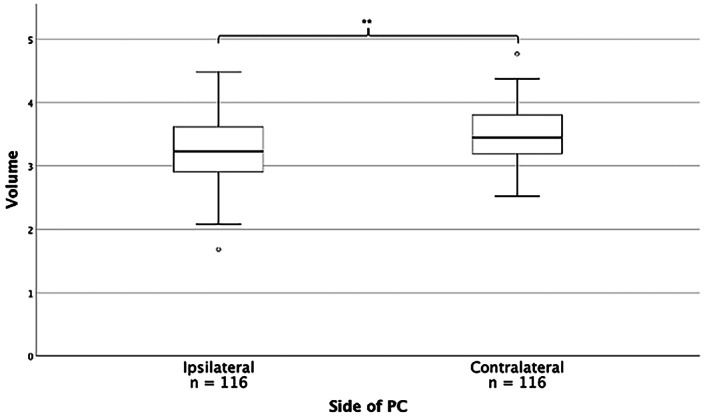
PC volumes in patients with temporal lobe epilepsy with hippocampal sclerosis. PC volume normalised by ICV comparing ipsilateral and contralateral side in relation to hippocampal sclerosis. **p < .001. For ease of reading, values were multiplied by 10^4^.

In TLE and HS (TLE + HS, *n* = 116), the average PC volume was 504 ± 69.6 mm^3^ (CV 14%), and ICV‐corrected PC volume was 3.32 ± 0.46 (CV 14%). The PC ipsilateral to HS was 7% smaller than the contralateral PC (489 vs. 523 mm^3^, *t* = −3.69, *df* = 230, *p* < .001; ICV‐corrected volume 3.26 vs. 3.48, *t* = −3.59, *df* = 230, *p* < .001; Figure 4). For comparison of PC volumes stratified by side of HS and in the 58 healthy young adults from the same study, compare Figure S3. No significant left–right difference was observed in the matched healthy controls (*n* = 58) from this group (*t* = −0.30, *df* = 114, *p* = .768), in line with the results from the Hammers Atlas Database group (cf. Section 3.1.1).

As expected, ipsilateral hippocampi and amygdalae were smaller on the side of the seizure focus (27% decrease of ICV‐corrected hippocampus volume: ipsilateral 12.15 ± 2.29 [CV 19%] vs. contralateral 16.69 ± 2.14 [CV 13%], *t* = −15.59, *df* = 230, *p* < .001; 5% decrease of ICV‐corrected amygdala volume: ipsilateral 9.06 ± 1.37 [CV 15%] vs. contralateral 9.57 ± 1.10 [CV 11%], *t* = −3.13, *df* = 230, *p* = .002; Figure S4).

#### 3.2.2 Patients with MCI and AD

No right–left difference of PC volumes was evident in patients with MCI (*t* = 1.55, *df* = 140, *p* = .123) or in matched healthy controls (*t* = 1.49, *df* = 92, *p* = .140). Patients with AD showed significant asymmetry (*t* = 2.37, *df* = 64, *p* = .021; Figure S5). In 33 patients with AD, the ICV‐corrected PC volume was 2.52 ± 0.48 (CV 19%) on the right and 2.25 ± 0.46 mm^3^ (CV 21%) on the left.

As right/left differences appear to be irrelevant for PC volume comparison between MCI, AD, and matched healthy controls, right and left PC volumes were combined for further analysis. The sum volume was 735 ± 141 mm^3^ (CV 19%) in patients with MCI (ICV‐corrected volume 4.91 ± 0.92, CV 19%), 674 ± 124 mm^3^ (CV 18%) in patients with AD (ICV‐corrected volume 4.77 ± 0.87, CV 18%) and 842 ± 111 mm^3^ (CV 13%) in healthy controls (ICV‐corrected volume 5.86 ± 0.80, CV 14%).

**FIGURE 5:**
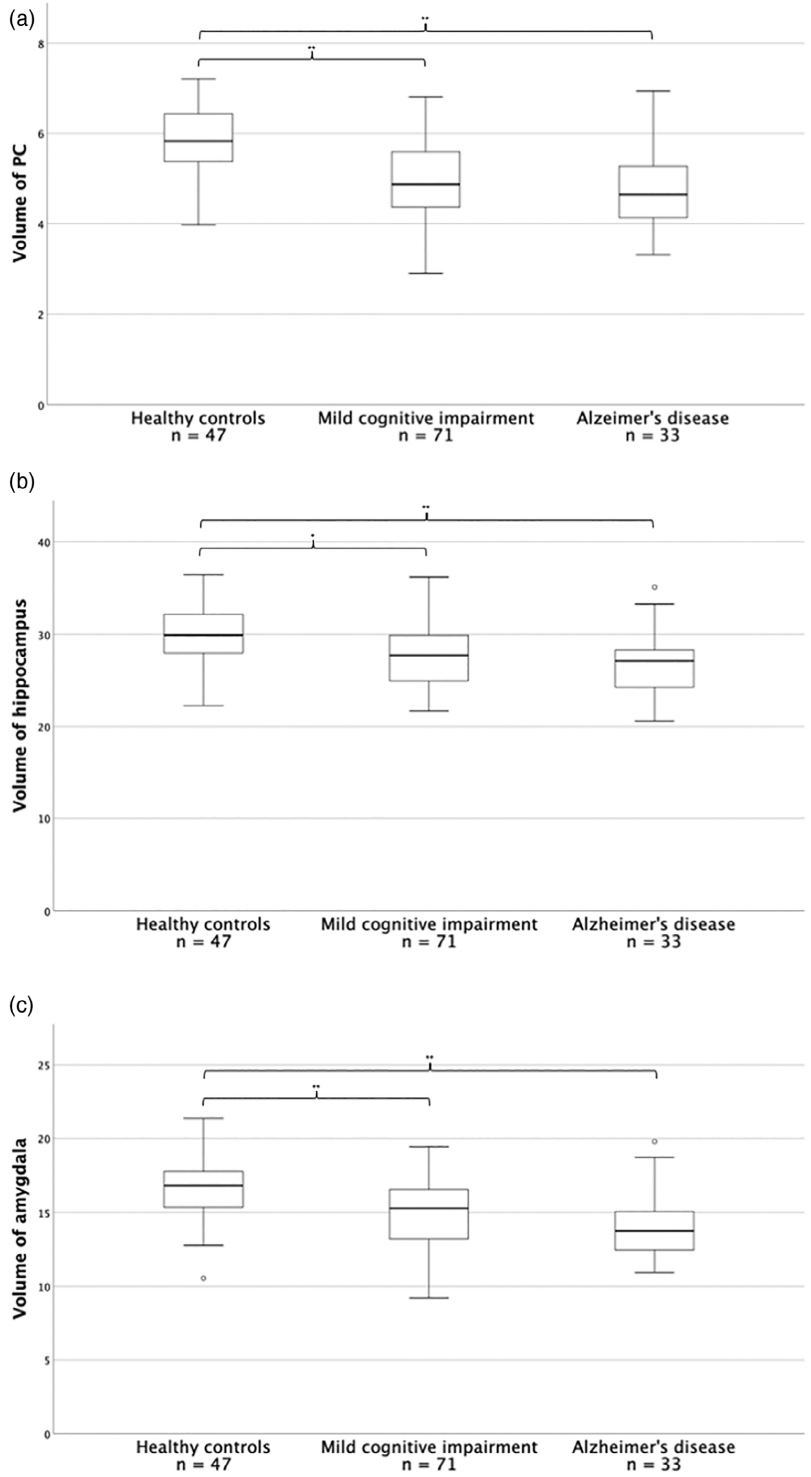
Volume decline in patients with mild cognitive impairment and Alzheimer's disease. Volumes normalised by ICV (right and left side) of PC (a), hippocampus (b), and amygdala (c), comparing healthy controls, patients with mild cognitive impairment, and patients with Alzheimer's disease. *p < .05. **p < .001. For ease of reading, values were multiplied by 10^4^.

Both in patients with MCI and with AD, the ICV‐corrected sum volume was smaller than in healthy controls (*F* = 21.09, *p* < .001; MCI: 16% volume discrepancy, *p* < .001; AD: 19% volume discrepancy, *p* < .001). No difference between MCI and AD was evident (Figure 5a).

Patients with pMCI had lower mean PC volumes (corrected by intracranial volume) than patients with sMCI (Table 4), but this difference was not statistically significant (t = −1.29, *df* = 69, *p* = .20). While the association was in the expected direction, there was no significant association between PC volume and latency between patient enrollment and clinical progress into AD (*r* = .264, *p* = .12, *n* = 36). In a sub‐analysis for patients with pMCI, the ICV‐corrected volume was smaller than in healthy controls (F = 23.04, *df* = 2, *p* < .001; pMCI: 19% volume discrepancy, *p* < .001; AD: 19% volume discrepancy, *p* < .001). No difference between pMCI and AD was evident.

**TABLE 4:** PC volumes in patients with MCI and AD.

	Healthy participants (*n* = 47)	Patients with sMCI (*n* = 35)	Patients with pMCI (*n* = 36)	Patients with AD (*n* = 33)
Right + left PC				
Volume in mm^3^ (CV)	842 (13%)	766 (19%)	705 (19%)	674 (18%)
ICV‐corrected volume (CV)	5.85 (14%)	5.05 (19%)	4.77 (18%)	4.77 (18%)

*Note*: Mean volumes (right and left side) and mean volumes normalised by ICV (right and left side) of PC in healthy controls, patients with stable mild cognitive impairment (sMCI), patients with progressive mild cognitive impairment (pMCI) and patients with Alzheimer's disease (AD). For ease of reading, values for ICV‐corrected volumes were multiplied by 10^4^.

As expected, hippocampal and amygdalar volumes were smaller (hippocampus: *F* = 9.46, *p* < .001; amygdala: *F* = 14.63, *p* < .001) in patients with MCI and AD than in healthy controls. Notably, atrophy was less pronounced in hippocampus and amygdala than in PC (Figure 5b,c). Hippocampal volume was 7% smaller in MCI (*p* = .002) and 10% smaller in AD (*p* < .001). Amygdalar volume was 11% smaller in MCI (*p* < .001) and 15% smaller in AD (*p* < .001).

## 4 DISCUSSION

We propose a manual segmentation protocol for delineating the human piriform cortex on MR images. We updated the Hammers Atlas Database with 30 instances of such segmentations and validated them via a four‐pronged approach: by ascertaining reliability and objectivity of the procedure in health and disease, by demonstrating feasibility of automatic PC segmentation when using the segmented images as training data, and by showing morphometric correlates of disease processes (temporal lobe epilepsy, Alzheimer's disease, and mild cognitive impairment) in the PC. Availability of the protocol, the segmentations, and the automatic segmentation procedure (MAPER) facilitates independent reproduction and replication of our work and enables users of the resources we share to conduct PC morphometry studies on their own imaging data.

Automatic segmentation of PC in healthy controls was accurate. Applied to patients with TLE with HS and to patients with AD, automatic segmentation of the PC yielded expected results, validating our method on a biological basis: PC volumes in TLE were lower ipsilateral to HS. In patients with MCI and in patients with AD, PC volumes were smaller compared to matched healthy controls. To our knowledge, this is the first study demonstrating PC atrophy in early (MCI) stages of AD.

The proposed manual PC delineation protocol can be applied reliably by inexperienced raters once they have received some training. Using the interrater measures of agreement (JC and volume discrepancy) as benchmarks, automatic delineation of PC was similarly accurate when applied to healthy participants in the Hammers Atlas Database. Bland–Altman plots showed volume differences within limits of agreement. Overlap measures such as JC decrease with surface‐to‐volume ratios (SVR) and increase with region volumes (Rohlfing et al., 2004). With respect to SVR and region volume, overlap between manual and automatic segmentations of PC was comparable to similarly anisotropic structures like the temporal horn of the lateral ventricle (Yaakub et al., 2020).

In patients with TLE with HS, the agreement between automatic and manual labels was as high as in the Hammers Atlas Database group. In patients with MCI and AD, it was lower than in the other two groups, but still satisfactory. In the presence of atrophy, the SVR will be higher, meaning that smaller and more dispersed JC values cannot be taken to indicate poorer segmentation accuracy.

Previous studies reported interrater analyses (Galovic et al., 2019, Data S1) with intraclass correlation coefficients or intrarater and interrater reliability assessment with Bland–Altman plots (Iqbal et al., 2022). In our study, we demonstrated that for our protocol there was also no significant volume difference in an intra‐ and interrater test using the Bland–Altman method. In addition, we conducted an interrater analysis with two additional independent raters. Going beyond volumetric comparison, we added overlap analyses using JC. This is important as even incongruent labels can have identical volumes.

For manual segmentation of PC, we mainly followed the work of Pereira et al. (2005), who used histological studies to validate defined anatomical landmarks for segmentation of the PC in MR images. The frontal part of the PC is less accurately defined by MR intensity gradients or landmarks than the temporal part. Pereira et al. (2005) excluded frontal PC from their definition, as they considered its borders in MR images insufficiently distinct. Other studies (Borger et al., 2021; Galovic et al., 2019) did include the frontal part into their definitions of the PC, building on the work of Mai et al. (2008). Considering that the area tempestas, a part of the frontal PC, is a particularly epileptogenic region, we decided to include the frontal PC in our anatomical definition of the region. We terminated PC segmentation in the most rostral slice anterior to the first appearance of hippocampus to avoid any overlap with hippocampal regions and to reach higher reproducibility. Pereira et al. (2005) defined the opening of the hippocampal fissure to mark the most caudal image to be included. This discrepancy in segmentation protocols might limit comparability with other studies, but it only affects minor parts of the piriform cortex/cortical amygdala region, which are located adjacent to the hippocampus. Considering that these PC parts have been included in previous hippocampus segmentations and presumably have been incorporated in standard anterior temporal lobe resection, we felt it was reasonable to focus on the PC part clearly distinguished from the hippocampus (rostral part of temporal PC and frontal PC) as represented in our segmentation protocol.

We note that the study by Iqbal et al. (2022) reported significantly larger volumes of the right PC than of the left PC in healthy controls. In contrast, this asymmetry was not evident in the current study and in the study of Pereira et al. (2005). This will be explained by the different termination of PC segmentation. Our study defines the last slice by first appearance of the hippocampus and Pereira et al. define the last slice by the opening of the hippocampal fissure (i.e., immediately adjacent landmarks), whereas Iqbal et al. described the complete fusion of the cerebral peduncle with the pons as the landmark for the caudal end of the PC. Considering the Yakovlevian torque with the right hemisphere extending further anteriorly, the latter PC definition with an external and remote landmark that is the same for both sides posteriorly will lead to a right/ left asymmetry.

The MAPER algorithm reproduces segmentations from a database of multiple atlases on an ‘unseen’ (not contained in the reference) target image. Algorithms of this class perform best when applied within‐cohort (as in the leave‐one‐out cross‐comparison). MAPER's design ensures relative robustness towards pathological change (Heckemann et al., 2010); nevertheless, pathology such as mesial temporal sclerosis usually implies suboptimal representation of the target image in the atlas data. When pathology alters a brain's anatomy, expert human raters are more flexible to adapt their visual interpretation of the target image. Accordingly, automatic segmentation labels tend to disagree more with the manual reference, the more the target image diverges from the ‘normal’ represented by the reference. This is reflected in the Bland–Altman plots: those generated on the Hammers Atlas Database group do not show proportional bias, whereas those for the TLE and ADNI groups do, that is, the volume loss tends to be underestimated compared with manual segmentation. This is a variant of regression towards the mean likely to occur when a training sample exhibits fewer extreme characteristics than the testing sample, for example, when the training (atlas, normal reference) sample represents a broader population than the testing (target, patient) sample. We note that volume bias did not prevent automatic detection of the abnormalities.

Leon‐Rojas et al. (2021) added manual PC segmentation to the GIF algorithm to create a template for automatic PC segmentation. The GIF algorithm performs well on small regions and brain regions which can only be delineated by grey‐scale gradients (Cardoso et al., 2015) and would hence be predicted to work well for PC. However, it is not publicly available so we could not add the PC as a region to be segmented by GIF.

In patients with TLE with HS, PC atrophy was seen on the same side as HS. The scale of atrophy in comparison with matched healthy controls was comparable between PC and amygdala, while the extent of atrophy in the ipsilateral hippocampus was considerably larger. This is in line with results of Pereira et al. (2005), who reported similar observations. Divergent definitions of PC (see above) preclude quantitative comparison of PC atrophy between the studies.

The PC is a key node within the epileptogenic network of focal epilepsies, particularly of TLE with HS (Laufs et al., 2011; Young et al., 2018). The important role of the PC in TLE was confirmed in studies by Galovic et al. (2019) and Borger et al. (2021), which demonstrated that resection of the PC predicts seizure freedom after TLE surgery.

While our biological findings in TLE corroborate those of earlier studies, we also generated novel results in the form of PC volumetry in MCI and AD. Atrophy of the PC was found in comparison with healthy controls. AD is known to affect olfaction in the sense of higher‐order deficits: patients progressively lose the ability to perceive odour quality, to discriminate between odours, and to identify them (Kesslak et al., 1988; Li et al., 2010). This indicates early involvement of higher‐order olfactory pathways that include the PC (Li et al., 2010), in congruence with findings in patients with MCI and AD who on functional MR imaging showed less activation than controls in the anterior piriform cortex during odour identification (Kjelvik et al., 2021).

The amygdala and hippocampus, direct neighbours of PC, are known to shrink during AD progression (Heckemann et al., 2011; Klein‐Koerkamp et al., 2014). PC volume loss was approximately twice as large as in the hippocampus, and also larger than in the amygdala. PC atrophy was similarly distinct in patients with MCI as in those with AD, suggesting PC atrophies early on in AD. PC volume may thus have a role as a novel biomarker for early AD stages.

The careful validation of automatic morphometry against manual segmentations is a strength of our study, as well as the large sample size for both pathologies examined. In Heckemann et al. (2011) we had performed equivalence testing for Jaccard overlaps averaged across 83 regions defined with MAPER and compared those with TOSTs (two one‐sided *t* tests, a type of equivalence test) across field strengths; this included three small regions in the vicinity of the PC (amygdala, hippocampus, and parahippocampal gyrus) which were also the most different between groups (controls, MCI, and AD). On this basis, we hypothesized that PC volumes would be equivalent between scanners, too. The results of the equivalence tests (TOSTs) in Table S1 show that the null hypothesis of scanner differences can be rejected for all comparisons. In conclusion, PC volumetry results were independent of the manufacturer of the scanning equipment.

A limitation is that the MR images in the Hammers Atlas Database had been acquired at 1.5 Tesla. While they are of good quality, certain anatomical landmarks are easier to identify on images acquired at higher field strengths, especially subtle correlates of the sulcus semiannularis, which marks the medial border of PC in rostral slices.

While empirically, intrarater and interrater agreement was strong, we found that identification of the limen insulae marking the most rostral slice on which PC was to be outlined could be difficult due to partial volume effects. Rater training mitigated these difficulties. While the method presented here will allow automatic determination of extent and volume of the PC, if manual verifications are desired, a corresponding training period should be envisaged.

## 5 CONCLUSION

The multi‐atlas MAPER pipeline and manual delineations of PC integrated into the Hammers Atlas Database enable reliable automatic delineation of PC in healthy adult participants as well as in patients with TLE and HS or AD. As expected, PC volumes are unilaterally smaller on the side of HS and bilaterally smaller in MCI and AD. We found that atrophy of the PC in MCI and AD occurred early and exceeded that of hippocampus and amygdala, pointing to a potential role as a biomarker. The possibility to obtain PC volumes at scale will now allow such biomarker and functional studies.

## CONFLICT OF INTEREST STATEMENT

The authors report no competing interests.

## Supporting information


**Figure S1.** Bland–Altman plots illustrating absolute difference in volume of piriform cortex (PC) between manual and automatic delineation in 30 healthy volunteers (Hammers Atlas Database). Blue lines show mean difference, red lines show standard deviation multiplied by 1.96. Right PC *r* = 0.386, *p* = 0.035; Left PC *r* = 0.272, *p* = 0.146.
**Figure S2.** Bland–Altman plots illustrating relative difference in volume of piriform cortex (PC) between manual and automatic delineation **(A)** in 20 patients with temporal lobe epilepsy with hippocampal sclerosis (*r* = 0.594, *p* < 0.001) and **(B)** in 20 patients with Alzheimer's disease (*r* = 0.481, *p* = 0.002). 40 data points are present in total as right and left PC are displayed together. Blue lines show mean difference, red lines show standard deviation multiplied by 1.96.
**Figure S3.** Volume of PC in patients with temporal lobe epilepsy with hippocampal sclerosis and healthy controls corrected by intracranial volume compared between right and left side of PC. HS: hippocampal sclerosis; PC: piriform cortex. *p < .05. For ease of reading, values were multiplied by 10^4^.
**Figure S4.** Volume of hippocampus and amygdala in patients with temporal lobe epilepsy with hippocampal sclerosis corrected by intracranial volume compared between ipsilateral and contralateral side in relation to side of hippocampal sclerosis. * p < 0.05; ** p < 0.001. For ease of reading, values were multiplied by 10^4^.
**Figure S5.** PC volume corrected by intracranial volume in healthy controls, patients with mild cognitive impairment (MCI) and patients with Alzheimer's disease (AD). PC: piriform cortex. * p < 0.05. For ease of reading, values were multiplied by 10^4^.
**Table S1.** Comparison of ICV‐corrected PC volume by scanner type for the ADNI cohort (n = 151). TOST procedures testing the equivalence of the volumetry results between scanner types, testing each PC side (left and right) and each scanner manufacturer (GE, n = 23; Philips, n = 50; Siemens, n = 78) against each other in pairs. We used [−0.7, 0.7] as the equivalence interval (~ 1 SD) and alpha = 0.05/6 = 0.008 (Bonferroni‐adjusted for 6 tests). The PC volumes of the ADNI cohort differentiated by different scanner types can be found in Table 3.

## Data Availability

All segmentations and data are publicly available (https://github.com/DavidSteinbart/PiriformCortex).
