# Supplementary material for: Automatic and manual segmentation of the piriform cortex: Method development and validation in patients with temporal lobe epilepsy and Alzheimer's disease
Source: Hum Brain Mapp. 2023 Apr 13;44(8):3196–209. doi: 10.1002/hbm.26274 (PMC10171523; doi:10.1002/hbm.26274)
Supplement: Supplementary file 1 — Figure S1. Bland–Altman plots illustrating absolute difference in volume of piriform cortex (PC) between manual and automatic delineation in 30 healthy volunteers (Hammers Atlas Database). Blue lines show mean difference, red lines show standard deviation multiplied by 1.96. Right PC r = 0.386, p = 0.035; Left PC r = 0.272, p = 0.146. Figure S2. Bland–Altman plots illustrating relative difference in volume of piriform cortex (PC) between manual and automatic delineation (A) in 20 patients with temporal lobe epilepsy with hippocampal sclerosis (r = 0.594, p < 0.001) and (B) in 20 patients with Alzheimer's disease (r = 0.481, p = 0.002). 40 data points are present in total as right and left PC are displayed together. Blue lines show mean difference, red lines show standard deviation multiplied by 1.96. Figure S3. Volume of PC in patients with temporal lobe epilepsy with hippocampal sclerosis and healthy controls corrected by intracranial volume compared between right and left side of PC. HS: hippocampal sclerosis; PC: piriform cortex. *p < .05. For ease of reading, values were multiplied by 104. Figure S4. Volume of hippocampus and amygdala in patients with temporal lobe epilepsy with hippocampal sclerosis corrected by intracranial volume compared between ipsilateral and contralateral side in relation to side of hippocampal sclerosis. * p < 0.05; ** p < 0.001. For ease of reading, values were multiplied by 104. Figure S5. PC volume corrected by intracranial volume in healthy controls, patients with mild cognitive impairment (MCI) and patients with Alzheimer's disease (AD). PC: piriform cortex. * p < 0.05. For ease of reading, values were multiplied by 104. Table S1. Comparison of ICV‐corrected PC volume by scanner type for the ADNI cohort (n = 151). TOST procedures testing the equivalence of the volumetry results between scanner types, testing each PC side (left and right) and each scanner manufacturer (GE, n = 23; Philips, n = 50; Siemens, n = 78) against each other [file HBM-44-3196-s001.docx]

**Supplementary material for**

Automatic and manual segmentation of the piriform cortex: method development and validation in patients with temporal lobe epilepsy and Alzheimer’s disease

David Steinbart, Siti N. Yaakub, Mirja Steinbrenner, Lynn S. Guldin, Martin Holtkamp, Simon S. Keller, Bernd Weber, Theodor Rüber, Rolf A. Heckemann, Maria Ilyas-Feldmann**^†^,** Alexander Hammers**^†^*** for the Alzheimer’s Disease Neuroimaging Initiative

**^†^These authors contributed equally to this work.**

***corresponding author**

**Email:** alexander.hammers@kcl.ac.uk

**This file includes:**

Piriform cortex outlining protocol

Figures S1 to S5 and Table S1

Supplementary material references

**1. Piriform cortex outlining protocol**

| The piriform cortex and the cortical amygdaloid nuclei (cortical nuclei of the amygdala = anterior cortical nucleus, medial nucleus, nucleus of the lateral olfactory tract, olfactory amygdala, periamygdaloid cortex and posterior cortical nucleus) are grouped together as they are closely related anatomically and functionally (Pereira et al., 2005). They are subsumed under the term **piriform-cortical amygdala (PC)**  PC was outlined in T1-weightend coronal 0.9375 mm slices, in a rostral to caudal direction.  **Step 1**  1^st^ slice (most rostral slice) is defined by the appearance of **limen insulae** (based on histologic analysis of Goncalves Pereira et al. (Pereira et al., 2005).  Limen insulae is defined by the junction between the insula and the temporal lobe (Galovic et al., 2019), e.g. by the appearance of a white matter tracts connecting insula and temporal lobe (Insausti et al., 1998).  Temporal part  The delineation of temporal part of PC, follows the outlining protocol of Goncalves Pereira et al. (Pereira et al., 2005).  Frontal part  The delineation of frontal part of PC follows the outlining protocol of Galovic et al. (Galovic et al., 2019). | |
| --- | --- |
| 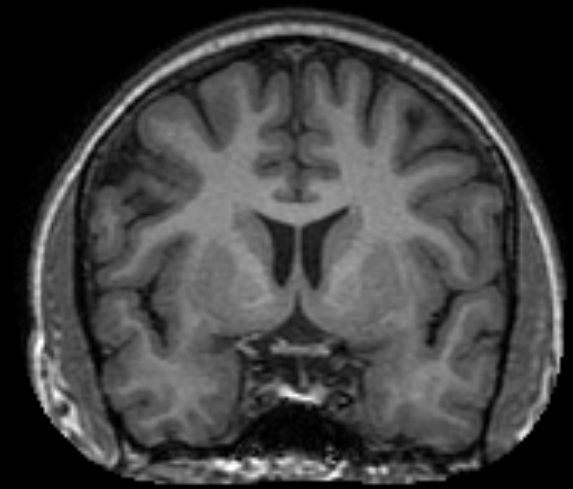  Image 1: Slice 120 of 198. Plain T1 (1.5 T) image of series “A06.nii” - Slice 120 of 198. (coronal 0.9375mm thick MR images). Image contrast adjusted manually. Interpolation mode “linear”. Imaging angle: Talairach anterior commissure (AC)-posterior commissure (PC) line  Limen insulae appears on the right side.  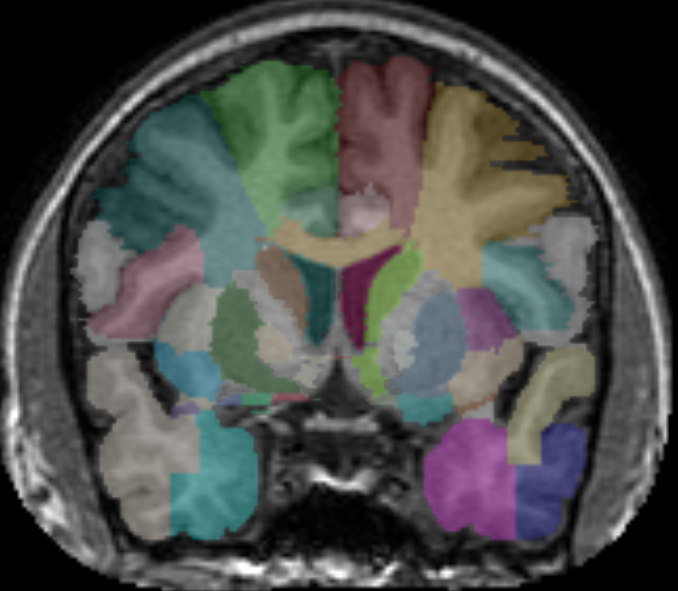  Image 2: Same slice with pre-existing labels of Hammers Atlas Datase (N30R124): Beginning temporal medial counter-clockwise: 5, 83, 21, 95, 93, 55, 73, 69 (list of labels can be found at the end of document)  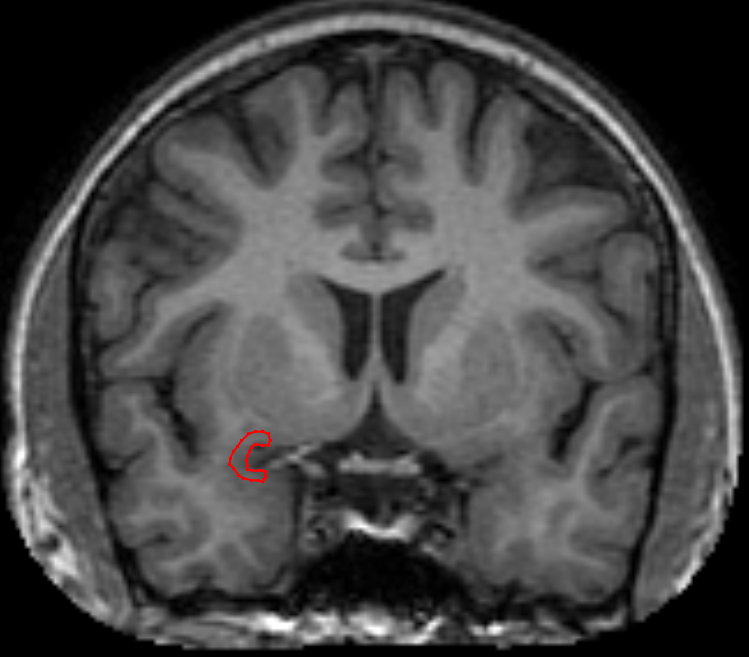  Image 3:  Same slice with delineation of most rostral part of PC. The outlining was performed with ITK-SNAP in “polygon mode”.  According to Goncalves Pereira et al. (Pereira et al., 2005), 1^st^ outline of temporal part of PC was drawn to delineate one-third of the distance from the endorhinal sulcus to the most convex point of the medial temporal lobe. Thickness was determined to be around 25% of the thickness of the perirhinal cortex (indicated with white dotted lines).  The lateral border was set by contrast to adjacent white matter tract.  For frontal part, we followed the algorithm of Galovic et al. and took account on Vaughan et al. (Vaughan & Jackson, 2014). We extended the frontal part of PC from the tip of the endorhinal sulcus, so that PC is eventually represented by a C-shaped delineation in first slice.  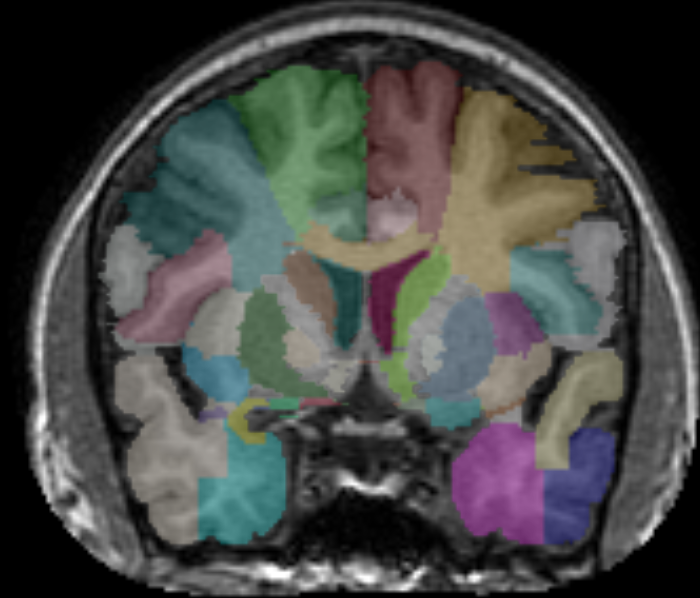  Image 4: New delineation of PC in yellow (Label No. 125) | |
| 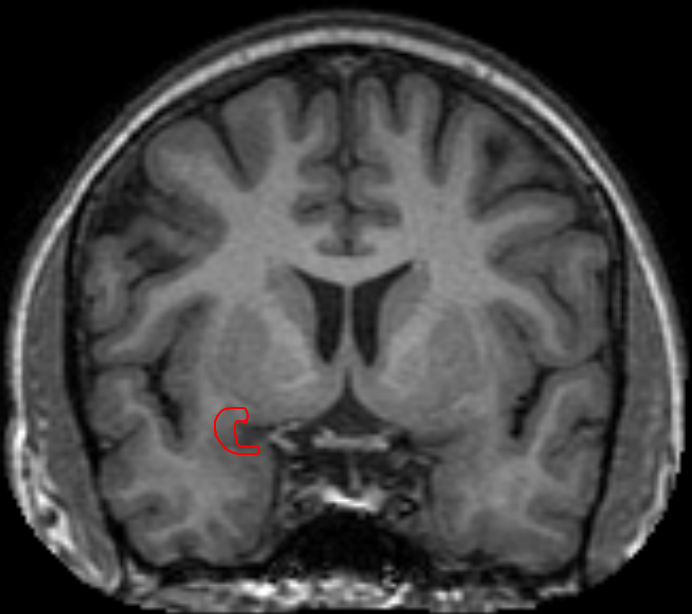 | **Step 2**  The temporal part is extended to occupy 30-50% of the distance of the endorhinal sulcus to most medial bump point of temporal lobe (=gyrus semilunaris) (Pereira et al., 2005). The thickness is kept to be around 25% of thickness of perirhinal cortex (thickness of perirhinal cortex is labelled with white line). For the lateral part, thickness is extended to the border of white matter.  The frontal part is kept constant (Galovic et al., 2019).  Image shows slice 119 of 198. |
| 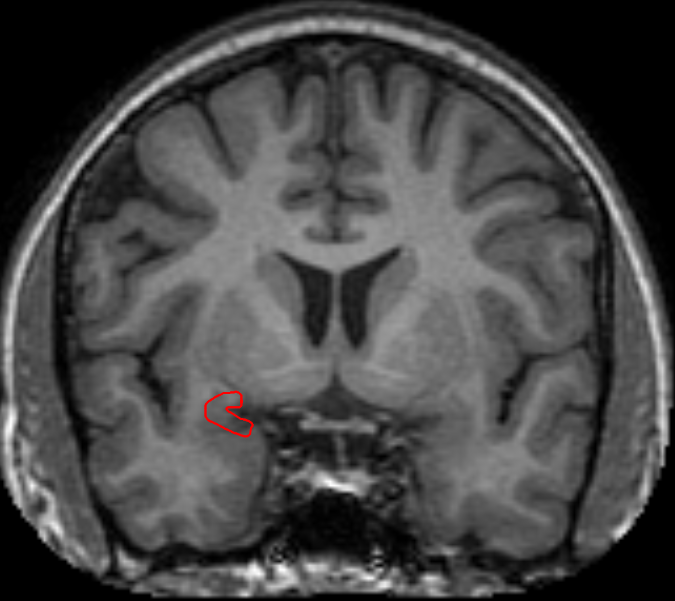 | **Step 3:**  The temporal part is extended to occupy 50-75% of the distance of the endorhinal sulcus to most medial bump point of temporal lobe (=gyrus semilunaris). The thickness is kept to around 25% of thickness of perirhinal cortex. For the lateral part, thickness is extended to the border of white matter.  The frontal part is kept constant, in this example the medial border of the frontal part of PC is well defined by a sulcus.  Image shows slice 118 of 198. |
| 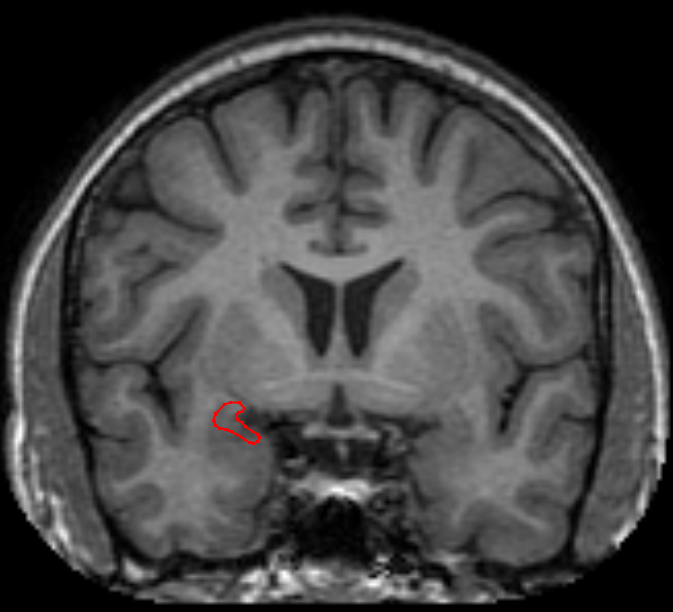 | **Step 4:**  The temporal part is extended to occupy around 75% of the distance of the endorhinal sulcus to most medial bump point of temporal lobe (=gyrus semilunaris). The thickness is kept to around 25% of thickness of perirhinal cortex. For the lateral part, thickness is extended to the border of white matter.  The frontal part is kept constant.  Image shows slice 117 of 198. |
| Image 1:  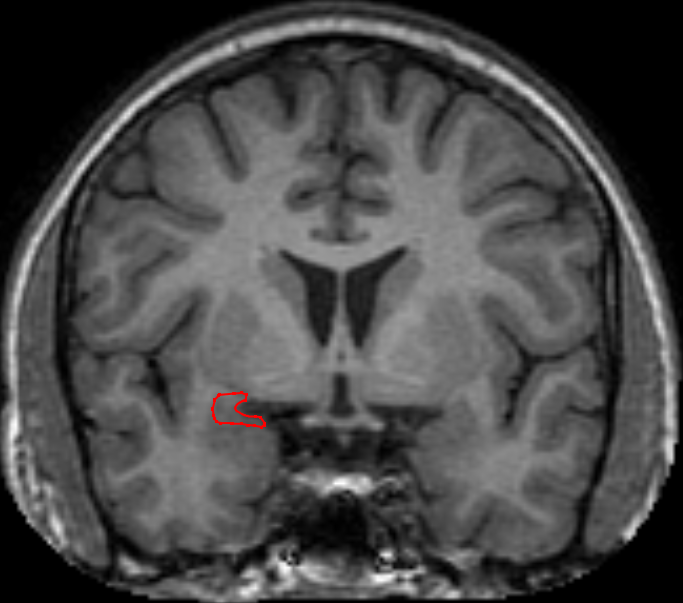  Image 2:  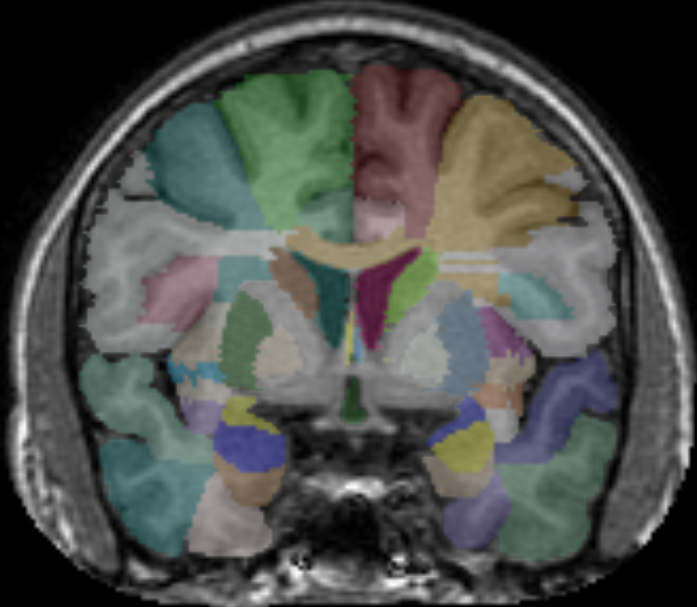 | **Step 5:**  This is the slice with the most rostral part of the amygdala (see image 2).  According to Goncalves Pereira et al. (Pereira et al., 2005), this slice is approximately the region where the most caudal section of the PC is present. Hence, in the following slices, only the cortical amygdaloid nuclei as part of the PC will be delineated.  In this slice (116 of 198), the entire cortex from the fundus of the endorhinal sulcus to the most medial point of the gyrus semilunaris is included. The sulcus semiannularis as medial border of PC can’t be clearly identified but could be estimated in the image.  Definition of a clear border between PC and amygdala is challenging, Goncalves Pereira et al. included “the entire thickness of the cortex […] in the PC” (Pereira et al., 2005).  Frontal part of PC was kept constant.  Image 2:  New delineation of PC in yellow (Label No. 125).  Note the amygdala in purple underneath. |
| Image 1:  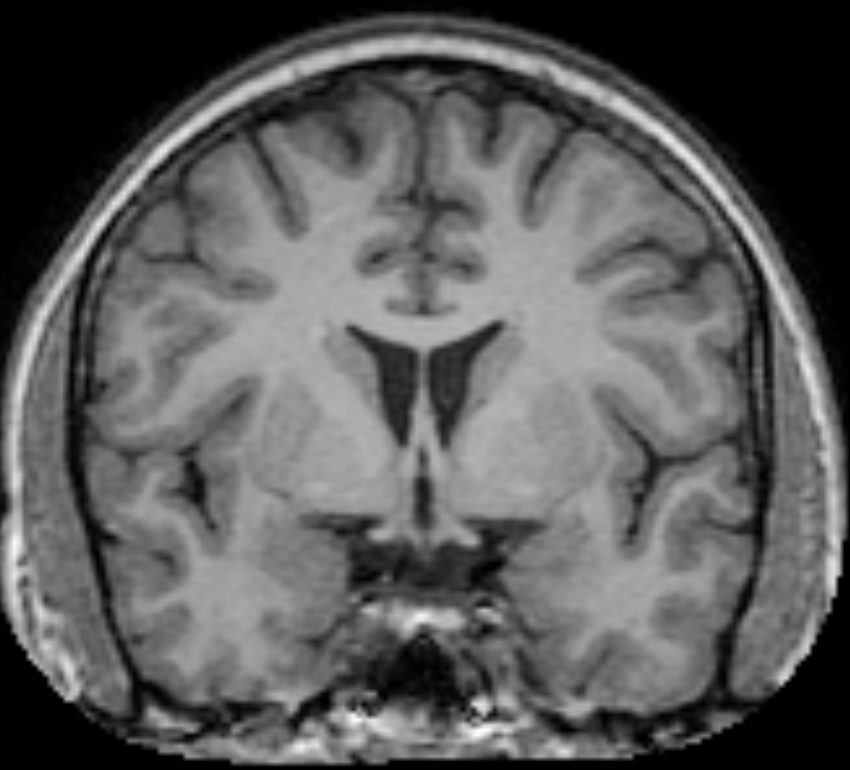  Image 2:  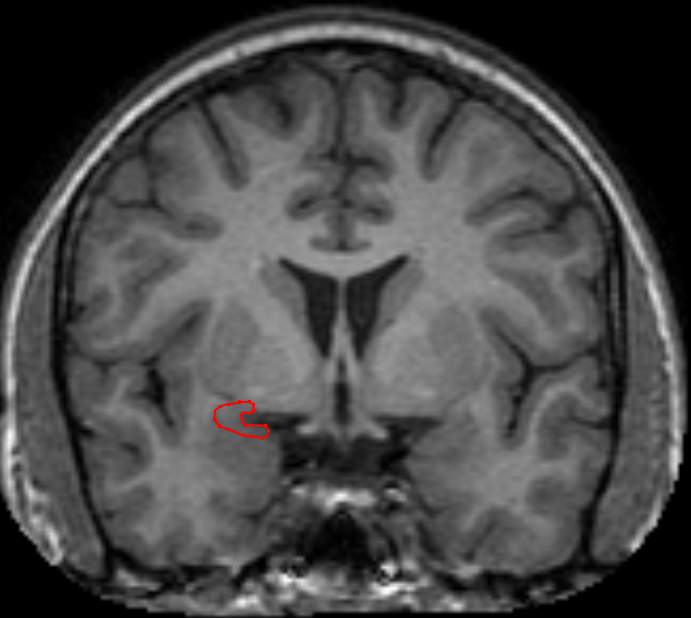 | **Step 6:**  Arrow indicates sulcus semiannularis (ssa) as medial border of PC, extension of ssa line serves as border line between PC and amygdala (dotted line).  Image shows slice 115 of 198.  Image 2:  Delineation of PC.  In this slice, the entire cortex from the fundus of the endorhinal sulcus to the most medial point of the gyrus semilunaris is included. The sulcus semiannularis as medial border of PC is probably present in this slice as indicated in image 1. |
| Image 1: Slice 114  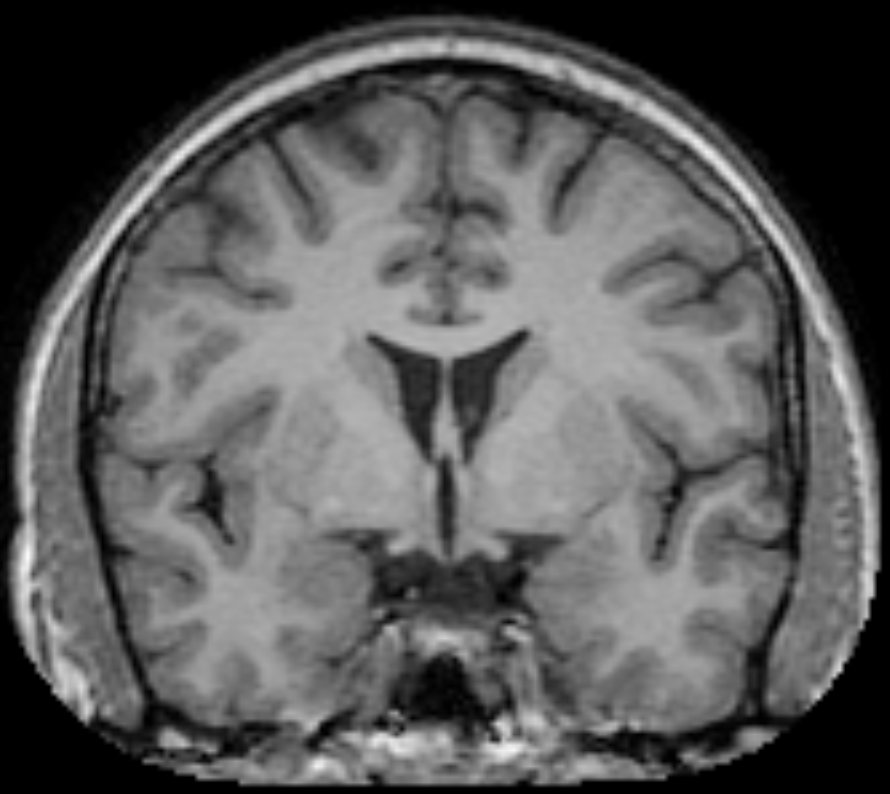  Image 2:  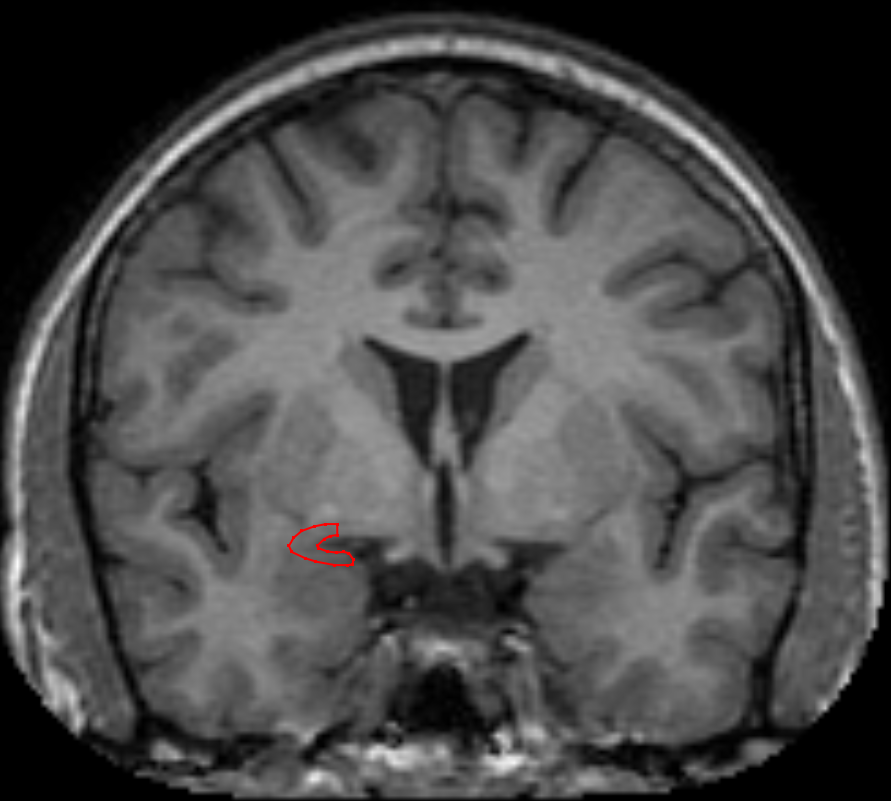  Image 3:  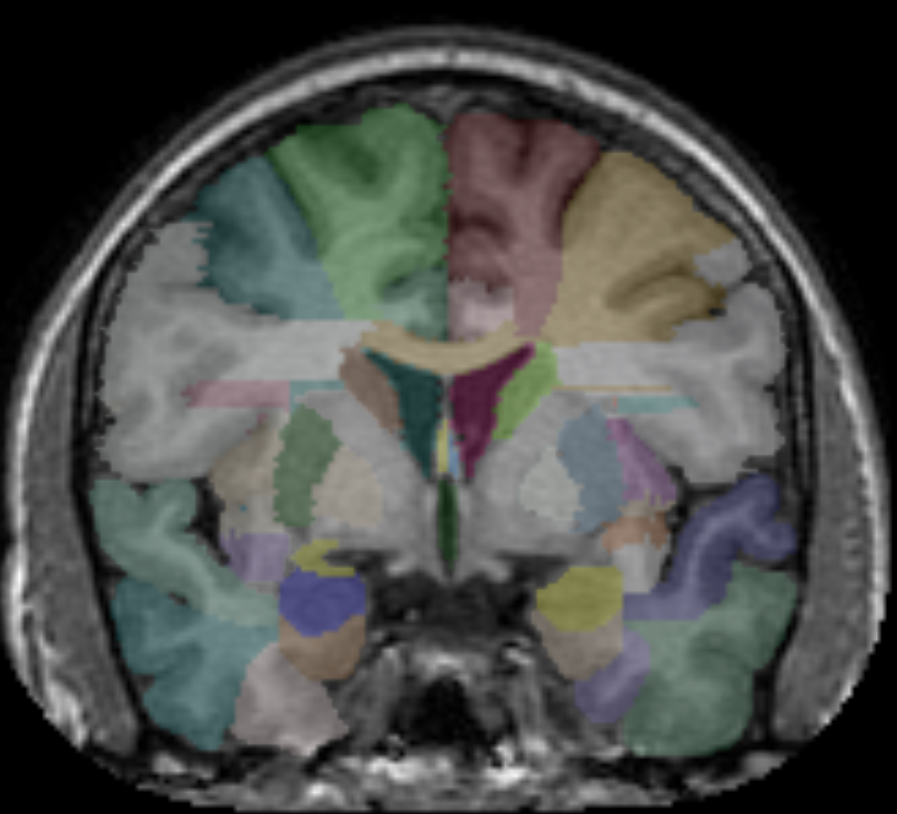 | **Step 7:**  Arrow indicates sulcus semiannularis (ssa) as medial border of PC.  Image shows slice 114 of 198  Image 2:  Delineation of PC.  In this slice, the entire cortex from the fundus of the endorhinal sulcus to the most medial point of the gyrus semilunaris is included. The sulcus semiannularis as medial border of PC is probably present in this slice and indicated in Image 1.  Definition of a clear border between PC and amygdala is a bit less challenging in this slice as a contrast in grey matter (extension of ssa-line) is considered as borderline.  Image 3:  New delineation of PC in yellow (Label No. 125).  Note the amygdala in purple underneath. |
| Image 1:  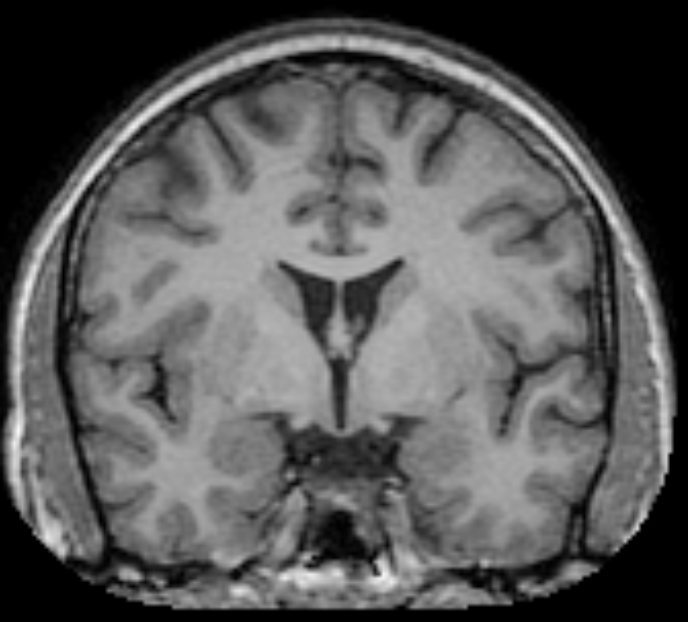  Image 2:  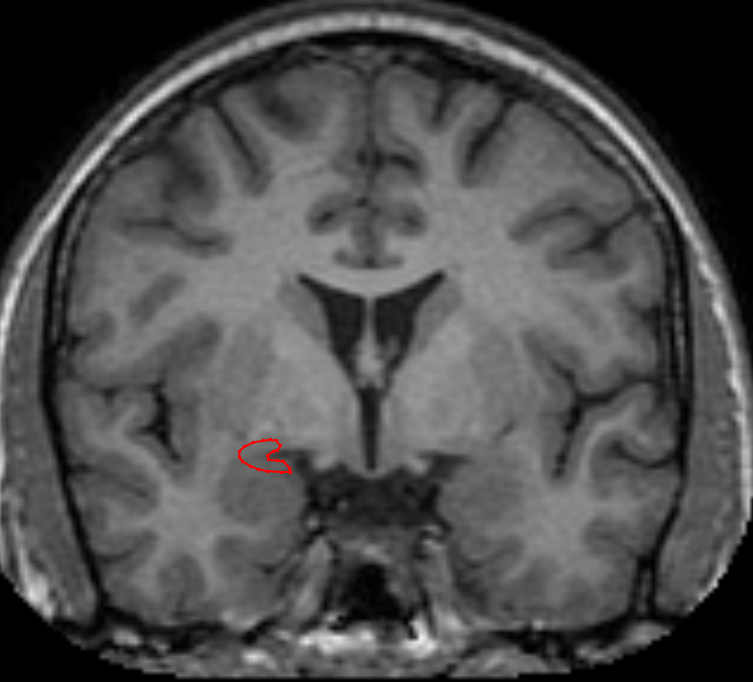  Image 3:  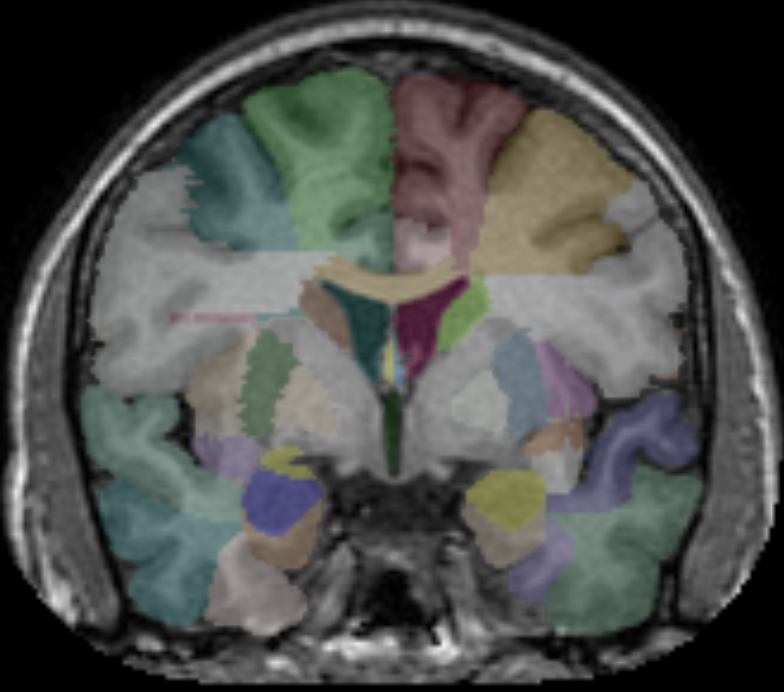 | **Step 8:**  Arrow indicates sulcus semiannularis (ssa) as medial border of PC.  Image shows slice 113 of 198.  Image 2:  Delineation of PC.  In this slice, the entire cortex from the fundus of the endorhinal sulcus to the most medial point of the gyrus semilunaris is included. The sulcus semiannularis as medial border of PC is probably present in this slice and indicated in Image 1.  Image 3:  New delineation of PC in yellow (Label No. 125).  Note the amygdala in purple underneath. |
| Image 1:  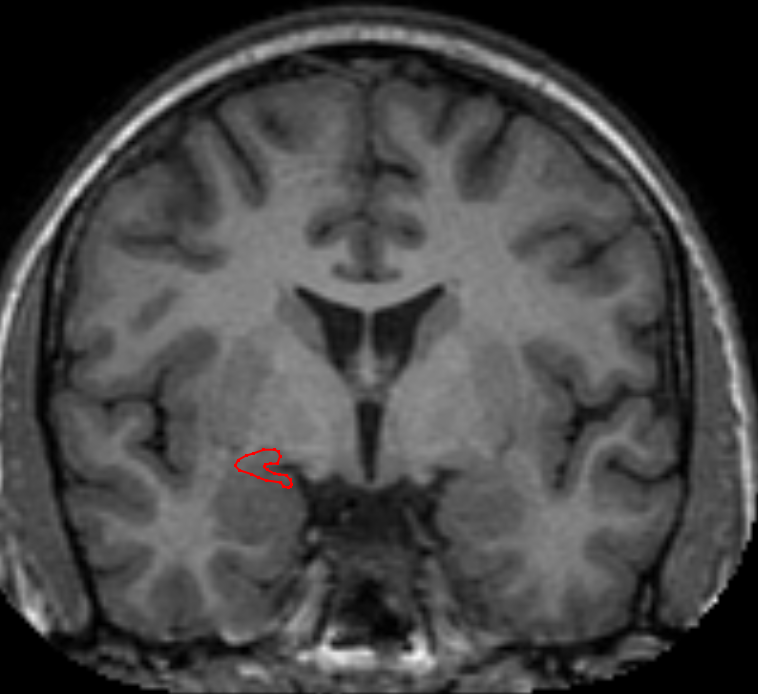  Image 2:  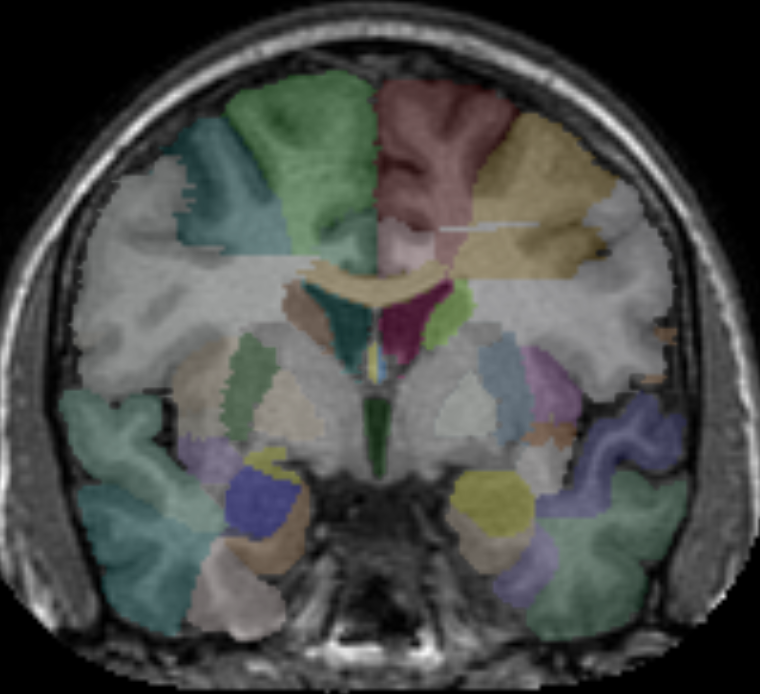 | **Step 9:**  Image shows slice 112 of 198.  In this slice, the entire cortex from the fundus of the endorhinal sulcus to the most medial point of the gyrus semilunaris is included.  The endorhinal sulcus becomes narrow at this time.  Definition of a border between PC and amygdala is done like in previous slice.  Image 2:  New delineation of PC in yellow (Label No. 125).  Note the amygdala in purple underneath.  The medial/ ventral border PC is analogue to border between amygdala (in purple) and parahippocampal and ambient gyrus (in brown).  This is the last slice before the first appearance of hippocampus. We decided to terminate the delineation PC within this slice to avoid an overlap with hippocampal regions.  As the hippocampus with the adjacent amygdalo-hippocampal area (AHA) is included into the standard anterior temporal lobe resection, from a clinical point of view, the AHA is not a region of interest. |

**List of labels of Hammers Atlas Database:**

1 "TL hippocampus R"

2 "TL hippocampus L"

3 "TL amygdala R"

4 "TL amygdala L"

5 "TL anterior temporal lobe medial part"

6 "TL anterior temporal lobe medial part"

7 "TL anterior temporal lobe lateral part"

8 "TL anterior temporal lobe lateral part"

9 "TL parahippocampal and ambient gyrus R"

10 "TL parahippocampal and ambient gyrus L"

11 "TL superior temporal gyrus middle part"

12 "TL superior temporal gyrus middle part"

13 "TL middle and inferior temporal gyrus"

14 "TL middle and inferior temporal gyrus"

15 "TL fusiform gyrus R"

16 "TL fusiform gyrus L"

17 "cerebellum R"

18 "cerebellum L"

19 "pons"

20 "insula posterior long gyrus L"

21 "insula posterior long gyrus R"

22 "OL lateral remainder occipital lobe L"

23 "OL lateral remainder occipital lobe R"

24 "CG anterior cingulate gyrus L"

25 "CG anterior cingulate gyrus R"

26 "CG posterior cingulate gyrus L"

27 "CG posterior cingulate gyrus R"

28 "FL middle frontal gyrus L"

29 "FL middle frontal gyrus R"

30 "TL posterior temporal lobe L"

31 "TL posterior temporal lobe R"

32 "PL angular gyrus L"

33 "PL angular gyrus R"

34 "caudate nucleus L"

35 "caudate nucleus R"

36 "nucleus accumbens L"

37 "nucleus accumbens R"

38 "putamen L"

39 "putamen R"

40 "thalamus L"

41 "thalamus R"

42 "pallidum L"

43 "pallidum R"

44 "corpus callosum"

45 "lateral ventricle excluding temporal horn R"

46 "lateral ventricle excluding temporal horn L"

47 "lateral ventricle temporal horn R"

48 "lateral ventricle temporal horn L"

49 "third ventricle"

50 "FL precentral gyrus L"

51 "FL precentral gyrus R"

52 "FL straight gyrus L"

53 "FL straight gyrus R"

54 "FL anterior orbital gyrus L"

55 "FL anterior orbital gyrus R"

56 "FL inferior frontal gyrus L"

57 "FL inferior frontal gyrus R"

58 "FL superior frontal gyrus L"

59 "FL superior frontal gyrus R"

60 "PL postcentral gyrus L"

61 "PL postcentral gyrus R"

62 "PL superior parietal gyrus L"

63 "PL superior parietal gyrus R"

64 "OL lingual gyrus L"

65 "OL lingual gyrus R"

66 "OL cuneus L"

67 "OL cuneus R"

68 "FL medial orbital gyrus L"

69 "FL medial orbital gyrus R"

70 "FL lateral orbital gyrus L"

71 "FL lateral orbital gyrus R"

72 "FL posterior orbital gyrus L"

73 "FL posterior orbital gyrus R"

74 "substantia nigra L"

75 "substantia nigra R"

76 "FL subgenual frontal cortex L"

77 "FL subgenual frontal cortex R"

78 "FL subcallosal area L"

79 "FL subcallosal area R"

80 "FL pre-subgenual frontal cortex L"

81 "FL pre-subgenual frontal cortex R"

82 "TL superior temporal gyrus anterior part"

83 "TL superior temporal gyrus anterior part"

84 "PL supramarginal gyrus L"

85 "PL supramarginal gyrus R"

86 "insula anterior short gyrus L"

87 "insula anterior short gyrus R"

88 "insula middle short gyrus L"

89 insula middle short gyrus R"

90 "insula posterior short gyrus L"

91 "insula posterior short gyrus R"

92 "insula anterior pole L"

93 "insula anterior pole R"

94 "insula anterior long gyrus L"

95 "insula anterior long gyrus R"

96 "medulla oblongata"

97 "midbrain"

98 "fourth ventricle"

99 "cerebral aqueduct"

100 "left cerebral white matter generated"

101 "left cerebral white matter generated"

102 "vermis"

103 "mamillary body R"

104 "mamillary body L"

105 "fornix R"

106 "fornix L"

107 "IFG pars opercularis R"

108 "IFG pars opercularis L"

109 "IFG pars triangularis R"

110 "IFG pars triangularis L"

111 "IFG pars orbitalis R"

112 "IFG pars orbitalis L"

113 "R Cerebellar Anterior Lobe"

114 "L Cerebellar Anterior Lobe"

115 "R Cerebellar Superior Posterior Lobe"

116 "L Cerebellar Superior Posterior Lobe"

117 "R Cerebellar Inferior Posterior Lobe"

118 "L Cerebellar Inferior Posterior Lobe"

119 "R Cerebellar Flocculonodular Lobe"

120 "L Cerebellar Flocculonodular Lobe"

121 "R Corpus Medullare"

122 "L Corpus Medullare"

123 "Subthalamic Nucleus R"

124 "Subthalamic Nucleus L"

125 "R piriform cortex"

126 "L piriform cortex"

**2. Figures and Tables**

**
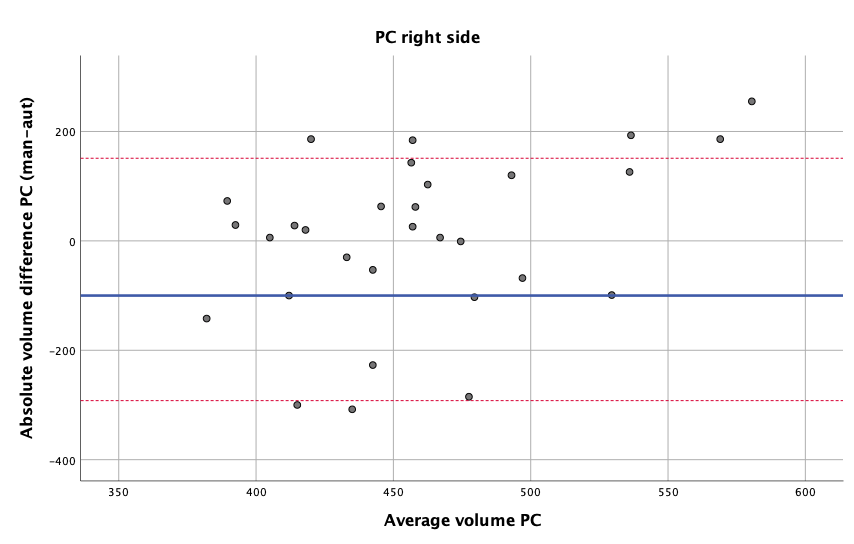
**


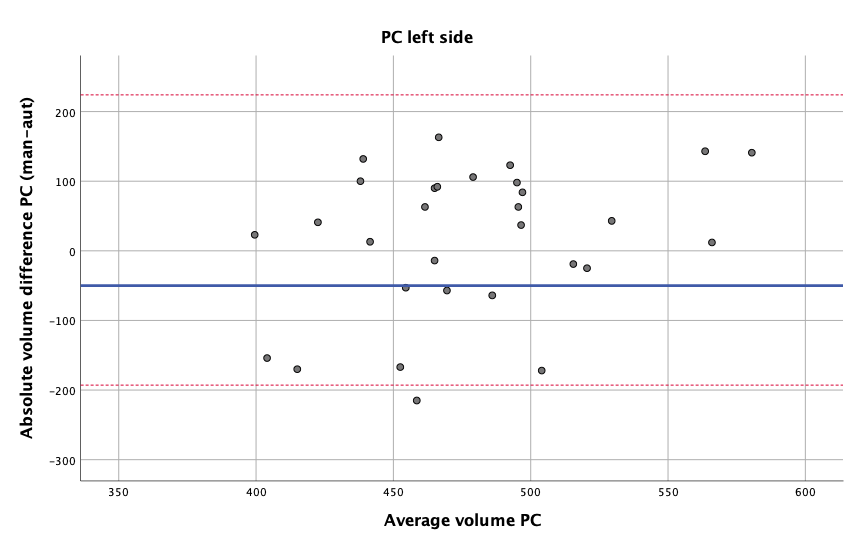


**Fig. S1.** Bland-Altman plots illustrating absolute difference in volume of piriform cortex (PC) between manual and automatic delineation in 30 healthy volunteers (Hammers Atlas Database). Blue lines show mean difference, red lines show standard deviation multiplied by 1.96.
Right PC *r* = 0.386, *p* = 0.035; Left PC *r* = 0.272, *p* = 0.146.

**(A)**


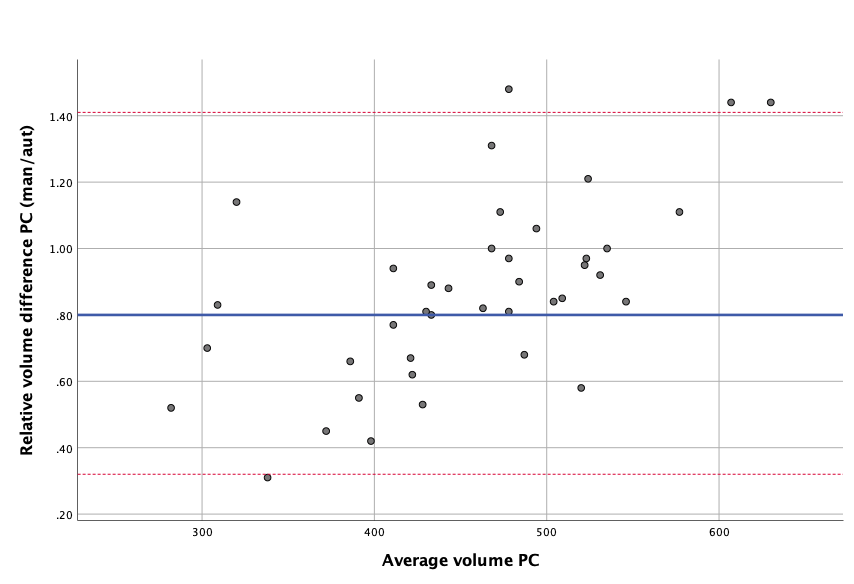


**(B)**


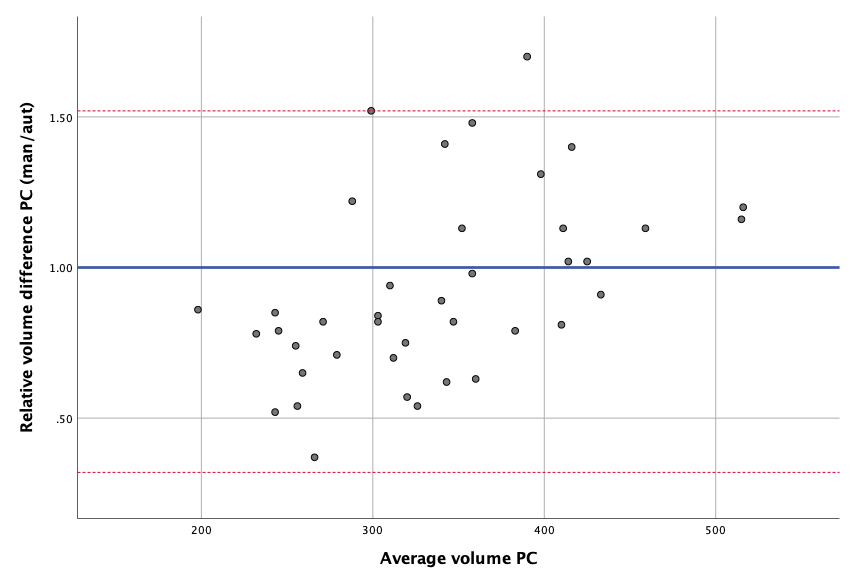


**Fig. S2.** Bland-Altman plots illustrating relative difference in volume of piriform cortex (PC) between manual and automatic delineation **(A)** in 20 patients with temporal lobe epilepsy with hippocampal sclerosis (*r* = 0.594, *p* < 0.001) and **(B)** in 20 patients with Alzheimer’s disease (*r* = 0.481, *p* = 0.002). 40 data points are present in total as right and left PC are displayed together. Blue lines show mean difference, red lines show standard deviation multiplied by 1.96.

*

*


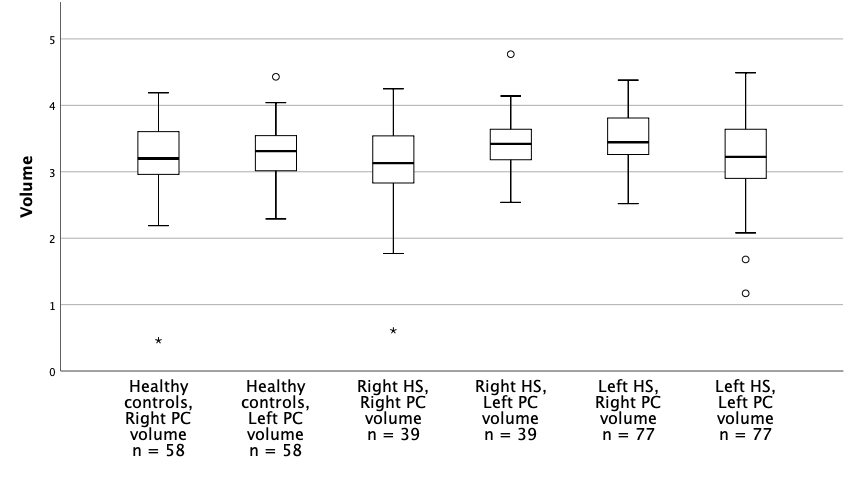


**Fig. S3.** Volume of PC in patients with temporal lobe epilepsy with hippocampal sclerosis and healthy controls corrected by intracranial volume compared between right and left side of PC.

HS: hippocampal sclerosis; PC: piriform cortex. * p < 0.05. For ease of reading, values were multiplied by 10^4^.

**

***

*

***

**
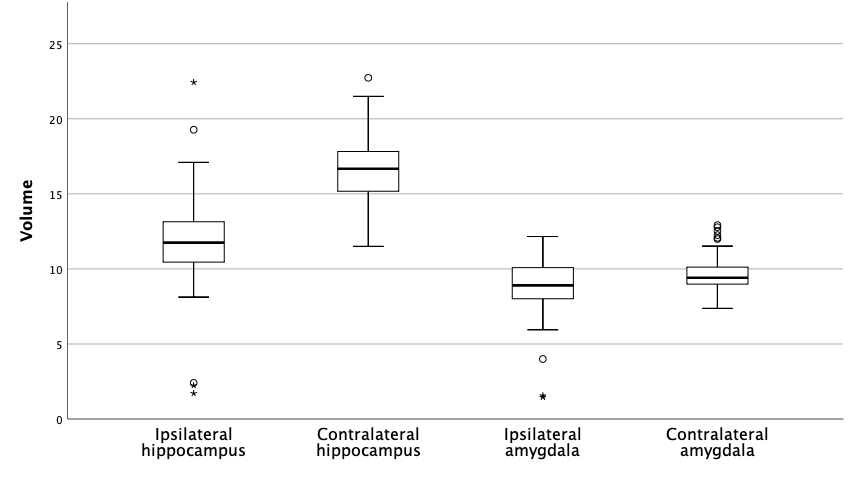
**

**Fig. S4.** Volume of hippocampus and amygdala in patients with temporal lobe epilepsy with hippocampal sclerosis corrected by intracranial volume compared between ipsilateral and contralateral side in relation to side of hippocampal sclerosis.

* p < 0.05; ** p < 0.001. For ease of reading, values were multiplied by 10^4^.

*


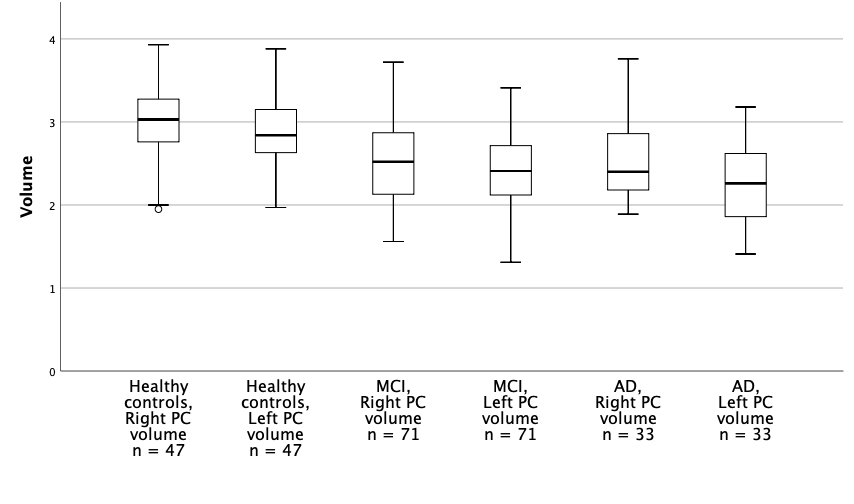


**Fig. S5.** PC volume corrected by intracranial volume in healthy controls, patients with mild cognitive impairment (MCI) and patients with Alzheimer’s disease (AD).

PC: piriform cortex. * p < 0.05. For ease of reading, values were multiplied by 10^4^.

**Table S1.** Comparison of ICV-corrected PC volume by scanner type for the ADNI cohort (n = 151).

TOST procedures testing the equivalence of the volumetry results between scanner types, testing each PC side (left and right) and each scanner manufacturer (GE, n = 23; Philips, n= 50; Siemens, n = 78) against each other in pairs. We used [-0.7, 0.7] as the equivalence interval (~ 1 SD) and alpha = 0.05/6 = 0.008 (Bonferroni-adjusted for 6 tests). The PC volumes of the ADNI cohort differentiated by different scanner types can be found in Table 3.

| **GE vs. Philips** | **TOST p-value 1** | **TOST p-value 2** | **Confidence Interval** |
| --- | --- | --- | --- |
| Right PC | 5.80 × 10^-4^ | 2.49 × 10^-8^ | [-0.554, 0.153] |
| Left PC | 1.59 × 10^-4^ | 4.52 × 10^-7^ | [-0.488, 0.244] |
| **GE vs. Siemens** |  |  |  |
| Right PC | 1.10 × 10^-4^ | 4.38 × 10^-7^ | [-0.478, 0.260] |
| Left PC | 6.16 × 10^-9^ | 5.45 × 10^-5^ | [-0.178, 0.476] |
| **Philips vs. Siemens** |  |  |  |
| Right PC | 1.10 × 10^-11^ | 5.07 × 10^-8^ | [-0.166, 0.349] |
| Left PC | 2.22 × 10^-16^ | 3.52 × 10^-5^ | [0.021, 0.521] |

**Supplementary material references**

Galovic, M., Baudracco, I., Wright-Goff, E., Pillajo, G., Nachev, P., Wandschneider, B., Woermann, F., Thompson, P., Baxendale, S., McEvoy, A. W., Nowell, M., Mancini, M., Vos, S. B., Winston, G. P., Sparks, R., Prados, F., Miserocchi, A., de Tisi, J., Van Graan, L. A., … Koepp, M. J. (2019). Association of Piriform Cortex Resection With Surgical Outcomes in Patients With Temporal Lobe Epilepsy. *JAMA Neurology*, *76*(6), 690–700. https://doi.org/10.1001/jamaneurol.2019.0204

Insausti, R., Juottonen, K., Soininen, H., Insausti, A. M., Partanen, K., Vainio, P., Laakso, M. P., & Pitkänen, A. (1998). MR volumetric analysis of the human entorhinal, perirhinal, and temporopolar cortices. *American Journal of Neuroradiology*, *19*(4), 659–671.

Pereira, P. M. G., Insausti, R., Artacho-Pérula, E., Salmenperä, T., Kälviäinen, R., & Pitkänen, A. (2005). MR volumetric analysis of the piriform cortex and cortical amygdala in drug-refractory temporal lobe epilepsy. *American Journal of Neuroradiology*, *26*(2), 319–332.

Vaughan, D. N., & Jackson, G. D. (2014). The Piriform Cortex and Human Focal Epilepsy. *Frontiers in Neurology*, *5*, 259. https://doi.org/10.3389/fneur.2014.00259
